# The non-canonical Wnt receptor Ror2 is required for cartilage cell polarity and morphogenesis of the craniofacial skeleton in zebrafish

**DOI:** 10.1242/dev.201273

**Published:** 2023-04-21

**Authors:** Daniel B. Dranow, Pierre Le Pabic, Thomas F. Schilling

**Affiliations:** ^1^ Department of Developmental and Cell Biology, University of California, Irvine, CA 92697, USA; ^2^ Department of Biology & Marine Biology, University of North Carolina, Wilmington, NC 28403, USA

**Keywords:** Zebrafish, Cartilage, Cell polarity, Non-canonical Wnt, Ror2

## Abstract

Non-canonical/β-catenin-independent Wnt signaling plays crucial roles in tissue/cell polarity in epithelia, but its functions have been less well studied in mesenchymal tissues, such as the skeleton. Mutations in non-canonical Wnt signaling pathway genes cause human skeletal diseases such as Robinow syndrome and Brachydactyly Type B1, which disrupt bone growth throughout the endochondral skeleton. Ror2 is one of several non-canonical Wnt receptor/co-receptors. Here, we show that *ror2^−/−^* mutant zebrafish have craniofacial skeletal defects, including disruptions of chondrocyte polarity. *ror1^−/−^* mutants appear to be phenotypically wild type, but loss of both *ror1* and *ror2* leads to more severe cartilage defects, indicating partial redundancy. Skeletal defects in *ror1/2* double mutants resemble those of *wnt5b^−/−^* mutants, suggesting that Wnt5b is the primary Ror ligand in zebrafish. Surprisingly, the proline-rich domain of Ror2, but not its kinase domain, is required to rescue its function in mosaic transgenic experiments in *ror2^−/−^* mutants. These results suggest that endochondral bone defects in ROR-related human syndromes reflect defects in cartilage polarity and morphogenesis.

## Teaser

**Summary:** This paper demonstrates essential roles for the non-canonical Wnt receptors, Ror1 and Ror2, in cartilage morphogenesis in zebrafish.

## INTRODUCTION

How cells coordinate their behaviors to form exquisitely shaped tissues is a fundamental question in developmental biology. Many signaling pathways have been implicated in the control of tissue shape across species, from flies to humans, notably planar cell polarity (PCP) signals propagated through cell-cell contact (Gray et al., 2011). Initially studied extensively in epithelia in *Drosophila*, core components of PCP pathways appear to function similarly in a variety of cell and tissue types in many animals, including vertebrate mesenchymal cells. Two pathways in particular, the non-canonical/β-catenin-independent Wnt (Wnt-PCP) and Fat-Dachsous (Dchs) cell polarity pathways, play crucial roles in such diverse cell types in vertebrates as epidermal cells in skin, neuronal cells in the cerebral cortex and hair cells of the inner ear (Vladar et al., 2009). In both pathways, tissue polarity depends upon asymmetric cell shapes and behaviors, as well as the distributions of cytoplasmic and membrane proteins involved in signaling. However, many questions remain as to the cell type-specific roles of different ligands and receptors in these pathways as well as the downstream cellular mechanisms that they regulate.

Defects in a growing list of PCP components in the Wnt-PCP and Fat-Dchs pathways cause a variety of human syndromes that affect skeletogenesis. For non-canonical Wnt signaling these include both autosomal dominant and recessive forms of Robinow syndrome (*ROR2*, *WNT5A*, *DVL1*, *DVL3*, *FZD2*, *NXN*) (Afzal et al., 2000; Lima et al., 2022; Person et al., 2010; van Bokhoven et al., 2000; White et al., 2018), Brachydactyly type B1 (*ROR2*) (Oldridge et al., 2000; Schwabe et al., 2000), Kleipert syndrome (Amor et al., 2019) and Simpson-Golabi-Behmel syndrome type 1 (*GPC4*) (Waterson et al., 2010). For Fat-Dchs signaling they include Van Maldergem (*DCHS1*, *FAT4*) (Cappello et al., 2013) and Hennekam (*FAT4*) (Alders et al., 2014) syndromes. Most of these syndromes include craniofacial abnormalities such as hypertelorism, broad and flat facial features, as well as skeletal dysplasias such as limb-shortening and brachydactyly. Why defects in these cell polarity pathways cause such specific skeletal phenotypes and precisely how the signals function to control polarized skeletal cell behaviors remain unclear.

Previously, we explored roles for Fat3a and Dchs2 in regulating cartilage morphogenesis in the embryonic zebrafish craniofacial skeleton (Le Pabic et al., 2014). We showed that both are required for ensuring chondrocyte polarity, including polarized morphogenetic movements involved in cell intercalation and chondrocyte stacking, as well as cartilage differentiation. Work by others has also shown essential roles for components of non-canonical Wnt signaling, including Wnt5b and Gpc4, in many of the same aspects of zebrafish craniofacial development and cartilage stacking (Sisson et al., 2015). Similarly, Wnt-PCP signaling regulates cartilage polarity in the developing growth plates of mammalian long bones (Kuss et al., 2014). These results suggest that Fat-PCP and Wnt-PCP pathways work together to control polarized cell behaviors that shape embryonic cartilages, and in some cases later endochondral growth zones, prefiguring the bones that replace their cartilage templates.

Receptor tyrosine kinase-like Orphan Receptor 2 (Ror2) is a member of the large and diverse receptor tyrosine kinase (RTK) superfamily, which includes the epidermal growth factor receptor (EGFR), fibroblast growth factor receptor (FGFR), platelet derived growth factor receptor (PDGFR), insulin growth factor receptor (IGFR), and Eph families of receptors, with a wide range of functions in development and disease. Formerly an orphan receptor, Ror2 joined the RTKs Ror1, Ryk and MuSK as confirmed Wnt receptors. Specifically, Ror1, Ror2 and Ryk directly bind certain classes of Wnts and help transduce β-catenin-independent, non-canonical Wnt signaling (Endo et al., 2015). Numerous studies in mammals point to Wnt5a as the preferred and primary ligand for Ror receptors. Ror2 can act as a Wnt5 receptor by forming homodimers (Liu et al., 2007, 2008), Ror1/Ror2 heterodimers (Paganoni et al., 2010; Yu et al., 2016) or as a co-receptor in a complex with Frizzled (Fz) receptors (Nishita et al., 2010; Oishi et al., 2003). Which mode of ligand-receptor binding prevails *in vivo* may be context-dependent. Downstream of ligand-receptor binding, some evidence suggests that activation of Ror2 signaling requires receptor autophosphorylation and that Ror2 phosphorylates downstream targets to transduce its signal (Endo et al., 2015). However, an increasing body of evidence suggests that Ror2, similar to RTKs Ror1 and Ryk, has no intrinsic kinase activity and should therefore be classified as a pseudokinase (Sheetz et al., 2020). Ror2 signaling is thought to regulate cytoskeletal dynamics, likely via dishevelled proteins (e.g. Dvl1, Dvl3) and the activation and action of a variety of downstream proteins including c-Jun N-terminal kinase (JNK) as well as members of the Rho-family of GTPases including Rac1, Cdc42, and RhoA (Stricker et al., 2017). Thus, it remains unknown precisely how the Ror receptors transduce Wnt-PCP signals to mediate cell polarity.

Here, we explore the functions of Ror receptors in non-canonical Wnt signaling in zebrafish skeletal development. By analyzing complete loss-of-function mutants in both Ror1 and Ror2, we show that Ror2 is required for cartilage cell polarity and stacking during jaw morphogenesis and that its function is partially redundant with Ror1 in these processes. We test requirements for several domains of Ror2 in its ability to rescue the mutant defects *in vivo* and show, for the first time, that the proline-rich domain (PRD) in the cytosolic portion of Ror2 is required for cartilage stacking and morphogenesis. Surprisingly, the kinase domain of Ror2 is dispensable in this context. In addition, Ror2 promotes focal adhesions in chondrocytes potentially important in cartilage stacking. Our results highlight conserved functions of Ror receptors in skeletogenesis and provide insights into developmental mechanisms by which Wnt5-Ror2 signaling controls polarized cell behaviors.

## RESULTS

### Ror2 is required for body axis elongation and craniofacial skeletal development

**Fig. 1.**
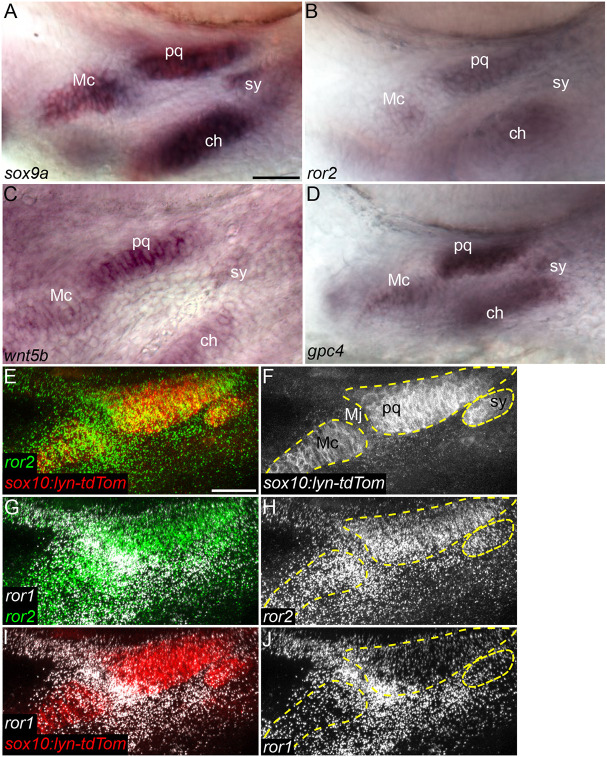
**Wnt cell polarity pathway genes are expressed in cartilage progenitors.** (A-D) *In situ* hybridization for *sox9a* (A), *ror2* (B), *wnt5b* (C) and *gpc4* (D) in 54 hpf wild-type (WT) embryos. Ventrolateral views of the mandibular and hyoid arches below the eye. (E-J) HCR for *ror1* and *ror2* in a 55 hpf WT *Tg(sox10-lyn-tdTomato)* (*sox10:lyn-tdTom*) embryo. *ror1* in white, *ror2* in green and *sox10:lyn-tdTom* in red (E,G,I). Grayscale (F,H,J). Panels E-J are *z*-projections. ch, ceratohyal cartilage; Mc, Meckel's cartilage; Mj, Meckel's joint; pq, palatoquadrate cartilage; sy, symplectic cartilage. Mc, pq and sy cartilages are outlined in dashed yellow line. Anterior to the left in all panels. Scale bars: 25 μm.

To investigate roles for Wnt-Ror signaling in zebrafish craniofacial development, we first examined the expression of several components of the non-canonical Wnt signaling pathway at embryonic stages of cranial cartilage differentiation. At 54 hours postfertilization (hpf), pharyngeal cartilage precursors actively stack and the first cartilage elements start to acquire their shapes (Le Pabic et al., 2014). Whole-mount *in situ* hybridization at this stage for *wnt5b*, as well *ror2* and *gpc4*, showed expression restricted to cartilage progenitors (Fig. 1A-D). Using fluorescent *in situ* hybridization chain reaction (HCR), we examined co-expression of *ror1*, *ror2* and a cartilage-specific *sox10:lyn-tdTom* transgene at both 54 and 72 hpf (Fig. 1E-J; Fig. S1). Whereas *ror2* was strongly expressed throughout skeletal condensations, *ror1* was expressed more in the surrounding mesenchyme, as well as strongly in the Meckel's-palatoquadrate joint.

**Fig. 2.**
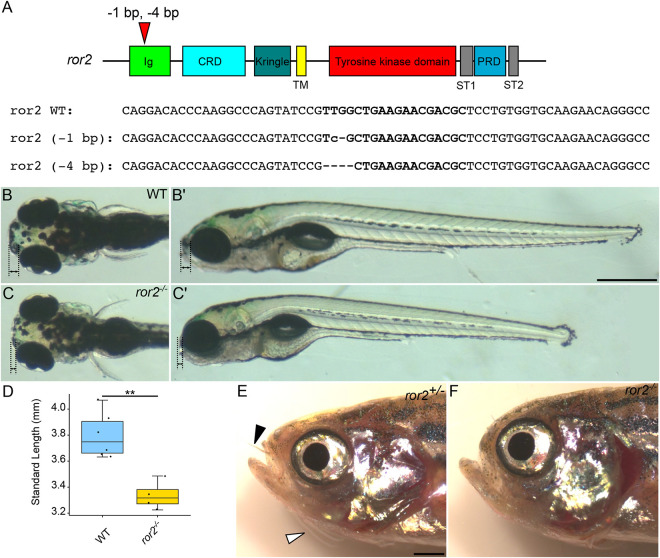
***ror2* mutants have craniofacial abnormalities and defects associated with disrupted cell polarity.** (A) Diagram of zebrafish *ror2* gene with domains annotated. Red triangle indicates gRNA target sites and the two alleles recovered. Bold text indicates gRNA target sites in the selected genomic DNA sequences. (B-C′) Representative images of 5 dpf wild type (WT) (B,B′) and *MZ-ror2* (C,C′) mutants. Dorsal (B,C) and lateral (B′,C′) views. Black dotted lines with double arrows indicate tissue anterior to the eyes. (D) Box plot comparing standard lengths of WT (*n*=6) and *ror2^−/−^* (*n*=4) embryos at 5 dpf. **P<0.01 (Wilcoxon rank-sum test). Box plot shows the median (middle bar) and first to third interquartile ranges (boxes); whiskers indicate 1.5× the interquartile ranges; dots indicate data points. (E,F) Phenotypically WT *ror2^+/−^* (E) and *ror2^−/−^* (F) adults. Black arrowhead indicates nasal barbel and white arrowhead indicates maxillary barbel. Ig, immunoglobulin-like domain; CRD, cysteine-rich domain; Kringle, Kringle domain; TM, transmembrane domain; ST1, serine-threonine domain 1; ST2, serine-threonine domain 2; PRD, proline-rich domain. Scale bars: 500 µm for B-C′; 1 mm for E,F.

To test requirements for Ror2, we used CRISPR-Cas9 mutagenesis to generate deletions in the genomic region corresponding to the Ig-like domain at the N terminus of the Ror2 receptor. We produced two mutant lines with deletions causing frameshifts that result in predicted loss-of-function alleles (Fig. 2A; Fig. S2B). In contrast to other non-canonical Wnt-PCP mutants [e.g. *wnt5b* (*pipetail*), *gpc4* (*knypek*), *vangl2* (*trilobite*)], *ror2^−/−^* mutants are adult-viable. Therefore, we incrossed homozygotes to generate maternal-zygotic *ror2*^−/−^ mutants (zygotic mutants that lack maternal contribution of wild-type (WT) RNA from the egg; *MZ-ror2*). Both alleles of *ror2*, either as *MZ-ror2* or zygotic *ror2^−/−^* appeared to be identical. For this reason, subsequent experiments and analyses were performed on *MZ-ror2^ir1093^* animals, hereafter referred to simply as *ror2^−/−^*. We found that *ror2^−/−^* mutant embryos were shorter than their WT counterparts at 5 days postfertilization (dpf), consistent with known roles for non-canonical Wnt signaling in convergent-extension (CE) of axial mesoderm and body axis elongation during gastrulation (Fig. 2C′,D) (Gray et al., 2011). *ror2^−/−^* mutant larvae also displayed a ‘hammerhead’ craniofacial phenotype, with reduced tissue anterior to the eyes (Fig. 2B-C′) (Schilling et al., 1996). Mutants in this category include *sox9a^−/−^*, the master regulator of cartilage differentiation (Yan et al., 2002). Adult *ror2^−/−^* mutants are shorter than WT or *ror2^+/−^*, with many craniofacial abnormalities (Fig. 2F; Fig. S3). Interestingly, we noticed that all *ror2^−/−^* mutants lacked both nasal and maxillary barbels, suggesting that Wnt5-Ror2 signaling is required for the formation and/or extension of these epidermal/sensory organs (Fig. 2E,F).

Because mutations in human *ROR2* can cause brachydactyly and *Ror2*^−/−^ knockout mice have shortened limbs, we examined the developing pectoral fin skeletons of zebrafish *ror2^−/−^* mutants. At 30 dpf, endoskeletal discs (ed) as well as the scapulocoracoid (sco) of the pectoral girdle appeared to be shorter and wider in *ror2^−/−^* mutants compared with WT (Fig. S4A,B). Individual chondrocytes of the sco, in particular, appeared to be rounder in mutants than in WT (Fig. S4C-F).

To confirm the reduction or loss of Ror2 protein in *ror2^−/−^* mutants, we performed anti-Ror2 antibody staining on embryos at 54 hpf using a monoclonal antibody raised against the C terminus of mouse Ror2. Whereas Ror2 was detected at cell membranes in developing cartilages including the Meckel's (Mc), palatoquadrate (pq) and, to a lesser extent, symplectic (sy) cartilages in WT (Fig. S5A-C), we were unable to detect Ror2 in skeletal progenitors in mutant animals (Fig. S5E-G), confirming that our mutant alleles are likely null.

### Ror2 is required for cartilage stacking and polarity

**Fig. 3.**
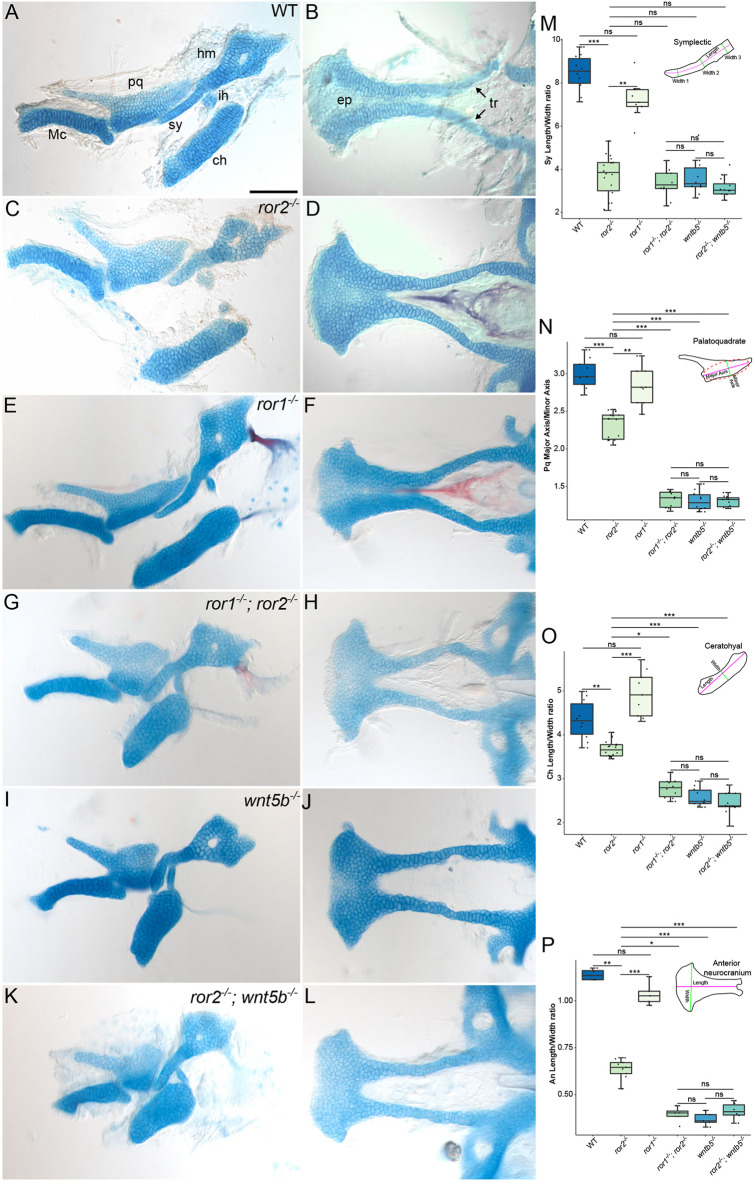
**Cartilage phenotypes in *ror1* and *ror2* mutants.** (A-L) Alcian Blue-Alizarin Red staining of craniofacial cartilages at 5 dpf. Lateral views of the upper and lower jaw cartilages (A,C,E,G,I,K) and cartilages of the anterior neurocranium (B,D,F,H,J,L). Representative images of cartilages from wild-type (WT; A,B), *ror2^−/−^* (C,D), *ror1^−/−^* (E,F), *ror1^−/−^; ror2^−/−^* (G,H), *wnt5b^−/−^* (I,J) and *ror2^−/−^; wnt5b^−/−^* (K,L) animals. Arrows point to trabecular cartilages. Anterior is to the left for all panels. (M-P) Quantifications of measured ratios: sy length-to-width (M); pq major axis-to-minor axis (N); ch length-to-width (O); An length-to-width (P). Diagrams of the sy cartilage, pq cartilage, ch cartilage and An indicating measured features are inset in panels M-P. *n*=10 WT, 9 *ror1^−/−^*, 18 *ror2^−/−^*, 10 *ror1^−/−^; ror2^−/−^*, 10 *wnt5b^−/−^* and 12 *ror2^−/−^; wnt5b^−/−^* sy cartilages for M. *n*=9 WT, 9 *ror1^−/−^*, 18 *ror2^−/−^*, 10 *ror1^−/−^; ror2^−/−^*, 10 *wnt5b^−/−^* and 12 *ror2^−/−^; wnt5b^−/−^* pq cartilages for N. *n*=10 WT, 9 *ror1^−/−^*, 18 *ror2^−/−^*, 10 *ror1^−/−^; ror2^−/−^*, 10 *wnt5b^−/−^* and 12 *ror2^−/−^; wnt5b^−/−^* ch cartilages for O. *n*=4 WT, 5 *ror1^−/−^*, 9 *ror2^−/−^*, 4 *ror1^−/−^; ror2^−/−^*, 5 *wnt5b^−/−^* and 6 *ror2^−/−^; wnt5b^−/−^* An for P. *P<0.05; **P<0.01; ***P<0.001 (Kruskal–Wallis test with post-hoc Dunn's test and Bonferroni correction). ns, not significant. Box plots show median (middle bar) and first to third interquartile ranges (boxes); whiskers indicate 1.5× the interquartile ranges; dots indicate data points. An, anterior neurocranium; ch, ceratohyal cartilage; ep, ethmoid plate; hm, hyomandibular cartilage; ih, interhyal cartilage; Mc, Meckel's cartilage; pq, palatoquadrate cartilage; sy, symplectic cartilage; tr, trabecular cartilage. Scale bar: 100 μm for A-L.

We next addressed roles for Ror2 in shaping individual cartilage elements, as well as in chondrocyte polarity within cartilages, by measuring their dimensions. Most craniofacial cartilages were shorter and wider in *ror2^−/−^* mutants than in WT counterparts (Fig. 3A-C), including those derived from the first and second pharyngeal arches; the pq, hyomandibular (hy), sy, and ceratohyal (ch) cartilages. We measured these differences for the sy cartilages (Fig. 3M), pq cartilages (Fig. 3N), ch cartilages (Fig. 3O) and anterior neurocrania (Fig. 3P) and found that compared with WT, mutant sy cartilages were on average 43% shorter and 36% wider, pq cartilages were 14% shorter and 10% wider, anterior neurocrania were 35% shorter and 14% wider, and ch cartilages were 12% shorter and 3.5% wider.

Ror1 is closely related to Ror2 and also expressed in craniofacial cartilage precursors, so we examined skeletal defects in *ror1^−/−^* as well as *ror1^−/−^; ror2^−/−^* double homozygous mutants (Fig. S2A). Similar to *ror2^−/−^*, *ror1^−/−^* mutants were homozygous viable, so we generated maternal-zygotic *ror1^−/−^* mutants and used these animals for all experiments, hereafter referred to as *ror1^−/−^* mutants. *ror1* expression in tissue surrounding developing cartilages, and strongly in the jaw joint, suggested that loss of Ror1 might specifically affect perichondrial or joint development (Fig. 1; Fig. S1). However, both zygotic and maternal-zygotic *ror1^−/−^* mutants appeared to be phenotypically WT, with no obvious defects in body axis elongation or facial features at either larval or adult stages. Consequently, the craniofacial cartilages of *ror1^−/−^* mutant larvae appeared to be phenotypically identical to WT (Fig. 3E,F,M-P). However, craniofacial cartilages in *ror1^−/−^; ror2^−/−^* double mutants appeared to be shorter and wider than *ror2^−/−^* mutants (compare Fig. 3C,D to G,H; M-P). Interestingly, this phenotype resembled that of zebrafish *wnt5b^−/−^* mutants (Fig. 3I,J). We also produced *ror2^−/−^*; *wnt5b^−/−^* double mutants, which closely resembled *wnt5b^−/−^* mutants (Fig. 3K,L,M-P), though their ch cartilages were somewhat shorter and wider (Fig. 3O). Together, these data support the hypothesis that Wnt5b is the ligand for Ror1/2 receptors in zebrafish. Though some *ror1^−/−^; ror2^−/−^* mutants survive to adulthood, the vast majority do not develop a swim bladder and die as larvae. Given the difficulty in raising these double homozygous mutants for experiments, all subsequent experiments and analyses focused on *ror2^−/−^* mutants.

**Fig. 4.**
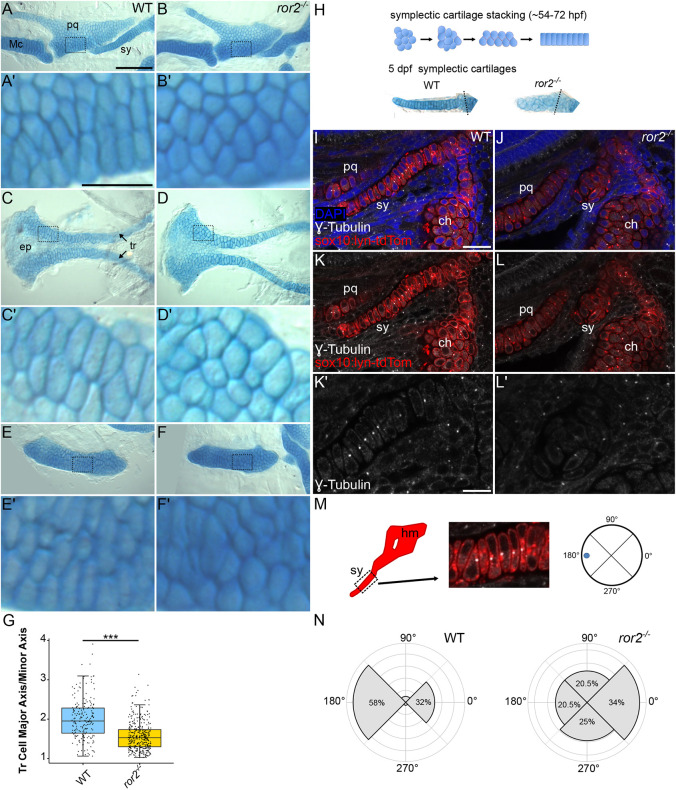
**Cell polarity is disrupted in *ror2* mutants.** (A-F′) Wild type (WT) and *ror2^−/−^* cartilages showing Palatoquadrate cartilages (A-B′), anterior neurocrania (C-D′) and ceratohyal cartilages (E-F′). Panels A′, B′, C′, D′, E′ and F′ are magnified views of the boxed regions in panels A, B, C, D, E and F, respectively. Arrows point to trabecular cartilages. (G) Quantifications of trabecular cell major axis-to-minor axis. ***P<0.001 (Wilcoxon rank-sum test). Box plot shows the median (middle bar) and first to third interquartile ranges (boxes); whiskers indicate 1.5× the interquartile ranges; dots indicate data points. *n*=195 WT and 379 *ror2^−/−^* tr cells. (H) Diagram of symplectic cartilage stacking and representative examples of 5 dpf Alcian Blue-stained symplectic cartilages. (I-N) Cell polarity measurements. Representative WT (I,K) and *ror2^−/−^* (J,L) *sox10:lyn-tdTomato* transgenic craniofacial cartilages stained with anti-γ-tubulin antibody in white. Panels I-L′ are a single slice of a *z*-stack. DAPI in blue, anti-γ-tubulin in white and *sox10:lyn-tdTomato* in red for I-L′. Panels K′ and L′ are magnified views of symplectic cartilages in K and L, respectively. (M) Diagram of how polarity was determined in symplectic cartilages. (N) Quantification of the distribution of the microtubule-organizing center (MTOC) in WT and *ror2^−/−^* symplectic cartilage cells. *P*<0.001 (Watson's two-sample test for homogeneity). ch, ceratohyal cartilage; ep, ethmoid plate; hm, hyomandibula; Mc, Meckel's cartilage; pq, palatoquadrate cartilage; sy, symplectic cartilage; tr, trabecular cartilage. Scale bars: 100 μm for A,B,C,D,E,F; 25 μm for A′,B′,C′,D′,E′,F′; 20 μm for I-L; 10 μm for K′,L′.

The observed differences in cartilage shape could reflect altered organization of the chondrocytes and/or changes in their shapes. On a cellular level, chondrocytes of cartilages of *ror2^−/−^* mutants appeared to be rounder than in WT (Fig. 4A-F′). Therefore, we quantified the dimensions of individual chondrocytes. For example, we measured the ratio between the long (length) and short (width) axes of cells of the trabecular (tr) cartilages (Fig. 4C′,D′) and found that chondrocytes in *ror2^−/−^* mutants were more round in shape, with an average length-to-width ratio of 1.57 (1.53 median) compared with 1.72 (1.95 median) of the narrow and long chondrocytes stereotypic of WT cartilages (Fig. 4G).

We hypothesized that defects in cell polarity underly the defects in cell and tissue shape of *ror2^−/−^* mutants. To examine the polarity of chondrocytes within craniofacial cartilages directly, we used the position of the centrosome/microtube organizing center (MTOC) as a readout (Le Pabic et al., 2014). For simplicity, we focused our analysis on the sy cartilage, as it is especially sensitive to polarity defects. The sy assembles via cell-cell intercalation in a process reminiscent of CE, which depends on PCP signaling in vertebrates (Kimmel et al., 1998) (Fig. 4H). Intracellular localization of MTOCs was used as a readout for cell polarity and its coordination across the many chondrocytes that make up the sy cartilage. To visualize MTOCs in sy, we performed anti-γ-tubulin antibody immunostaining on *ror2^−/−^* mutants and WT embryos at 72 hpf (Fig. 4I-L′). In WT, a vast majority of MTOCs are positioned anteriorly (along the anterior-posterior axis of the long axis of the cartilage element) in each chondrocyte, while the second-most common location is posterior in each cell (Fig. 4M,N). In contrast, in *ror2^−/−^* mutants, MTOCs were distributed almost equally amongst the dorsal, ventral, anterior and posterior directions in sy chondrocytes, with a bias towards the posterior (Fig. 4N). The loss of coordinated MTOC polarity correlates with the abnormal chondrocyte cell shape of *ror2^−/−^* mutants. Our results show that loss of Ror2 signaling not only disrupts the shapes of cartilages and the cells that form them, but the localization of intracellular components as reflected by the mislocalization of MTOCs.

### Loss of Ror2 alters focal adhesions in chondrocytes

**Fig. 5.**
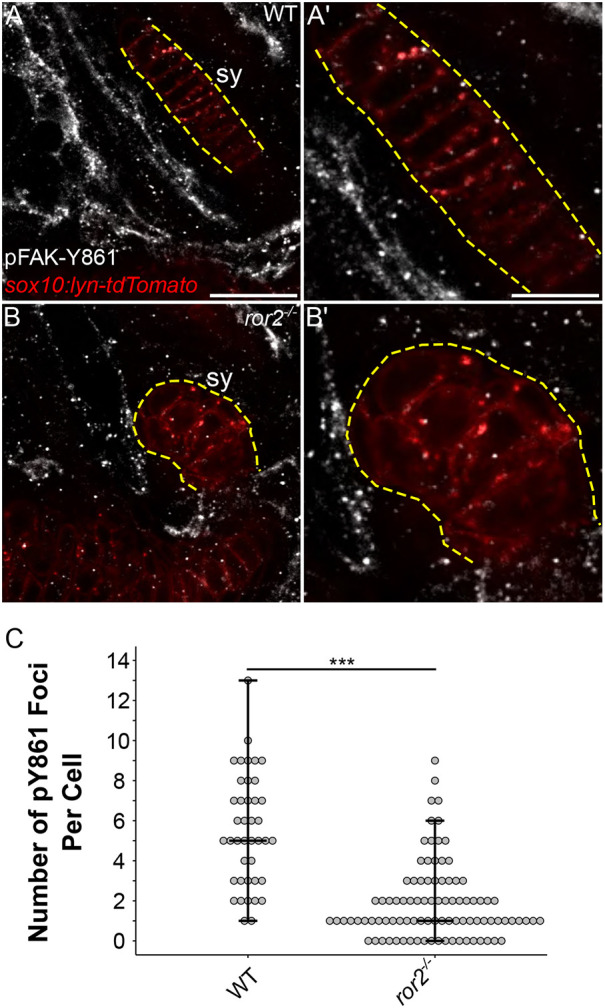
***ror2* chondrocytes have a reduced number of focal adhesions.** (A-B′) pFAK staining. Representative symplectic cartilages in WT (A) and *ror2^−/−^* (B) *sox10:lyn-tdTomato* transgenic fish at 3 dpf stained with anti-phospho-tyrosine 861 focal adhesion kinase antibody (pFAK-Y861) in white. Magnified views of symplectic cartilages in A′ and B′, respectively. *Sox10:lyn-tdTomato* chondrocytes in red. Panels A and B are a single slice of a *z*-stack. Dashed yellow lines delineate the symplectic cartilage visible in the selected slice. (C) Quantification of the number of pY861 foci per cell. Each point represents a single cell. Maximum, minimum and median values are depicted as crossbars on the dot plot. Sy, symplectic cartilage. ***P<0.001 (Wilcoxon rank-sum test). Scale bars: 20 μm for A and B; 10 μm for A′ and B′.

We next sought to gain insight into the cellular processes downstream of Ror2 receptor activation, which, when dysregulated, account for the loss of coordinated cell polarity in mutant chondrocytes. Regulation of cell-cell and cell-extracellular matrix (ECM) adhesion are important aspects of tissue morphogenesis. During cartilage morphogenesis, differentiating chondrocytes secrete extensive ECM defined early in resting and proliferating chondrocytes by the expression of *col2a1* and defects in the composition or integrity of the ECM cause well-known skeletal diseases/dysplasias (Krakow, 2015; Krakow and Rimoin, 2010). Focal adhesions (FAs) are adhesive protein complexes that link the actin cytoskeleton to the ECM through integrins (Itgs). Given the importance of ECM deposition and composition to cartilage morphogenesis, including cell polarity, and crosstalk between the cell and the surrounding ECM, we investigated whether FAs were affected in *ror2* mutant chondrocytes. To visualize FAs, we used an antibody to label phosphorylated focal adhesion kinase (pFAK) (Fig. 5A-B′). *ror2^−/−^* mutant chondrocytes in sy cartilages at 72 hpf had an average of 1.9 pFAK foci per cell (1 median) compared with 5.5 pFAK foci (5 median) in WT animals (Fig. 5C). Loss of pFAK foci indicate a reduction in focal contacts, which are necessary for proper Itg-mediated cell-cell interactions and migration (Stricker et al., 2017).

### Ror2 function in cartilage stacking requires Wnt-binding and proline-rich domains

**Fig. 6.**
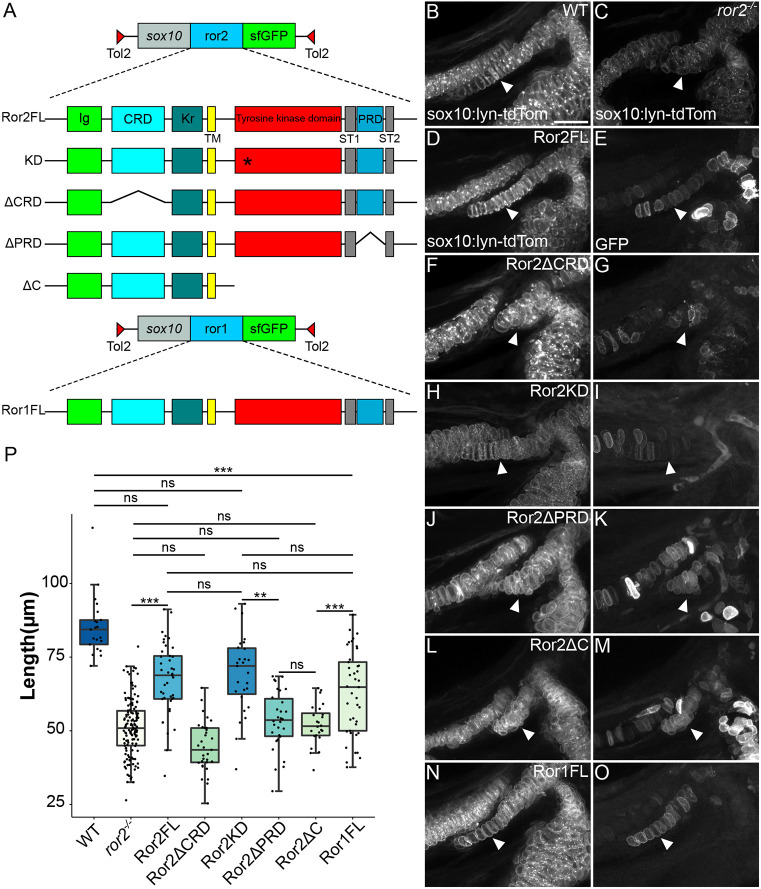
**Ror2 domain analysis reveals differential requirements for cartilage stacking.** (A) Schematic of *ror2* transgenic constructs. A *sox10* enhancer element drives expression of Ror2-SuperFolderGFP (sfGFP) fusions in a Tol2 vector. Constructs include full-length Ror2-sfGFP (Ror2FL), Ror2K509R-sfGFP (KD), Ror2ΔCRD-sfGFP (ΔCRD), Ror2ΔPRD-sfGFP (ΔPRD), Ror2ΔC-sfGFP (ΔC) and full-length Ror1 (Ror1FL). Asterisk indicates the location of the K509R mutation. (B,C) Confocal images of fluorescent cartilages at 3 dpf in WT (B) and *ror2^−/−^* (C) in a *Tg(sox10:lyn-tdTomato)* background. (D-O) Representative rescue construct mosaic transgenic cartilages in grayscale showing *sox10:lyn-tdTomato* only (D,F,H,J,L,N) or GFP only (E,G,I,K,M,O). White arrowheads indicate the symplectic cartilage. Panels B-O are z-projections. (P) Quantification of symplectic cartilage length, color-coded according to magnitude. **P<0.01; ***P<0.001 (Kruskal–Wallis test with post-hoc Dunn's test and Bonferroni correction). ns, not significant. Box plot shows the median (middle bar) and first to third interquartile ranges (boxes); whiskers indicate 1.5× the interquartile ranges; dots indicate data points. CRD, cysteine-rich domain; Ig, immunoglobulin-like domain; Kr, Kringle domain; PRD, proline-rich domain; ST1, serine-threonine domain 1; ST2, serine-threonine domain 2; TM, transmembrane domain. Scale bar: 20 μm for B-O.

Various functions for Ror receptors that either depend upon the cytoplasmic and kinase domains or act independently have been proposed. To investigate these proposed functions in Ror2 regulation of cartilage stacking and craniofacial morphogenesis, we explored the requirements for specific domains of the receptor to rescue the *ror2*^*−/−*^ craniofacial phenotype. The extracellular portion of Ror2 consists of an Ig-like domain (Ig), a Wnt-binding cysteine-rich domain (CRD), similar to a frizzled domain, and a Kringle (Kr) domain, which is thought to interact with Wnt regulatory proteins such as Dickkopf (Dkk), but the function of which in Ror receptors is largely unknown (Fig. 2A) (Endo et al., 2015; Mao et al., 2002). The transmembrane domain (TM) precedes the kinase domain and a proline-rich domain (PRD) flanked by two serine-threonine-rich domains (ST1 and ST2). We produced a variety of zebrafish Ror2-super folder GFP (sfGFP) fusion constructs, injected them into *ror2*^*−/−*^ mutants at the one-cell stage to produce mosaic transgenic individuals, and asked whether full-length or various truncated forms of Ror2 were able to rescue cartilage stacking. We focused our analysis on the sy cartilage owing to its simplicity and consistent stacking defects in mutants; rescue in transgenic mosaic animals was evaluated based on sy cartilage length. Transgenic constructs included full-length Ror2 (Ror2FL), Ror2 with a mutation predicted to render any RTK kinase domain inactive (kinase-dead mutation; Ror2KD), with the CRD deleted (Ror2ΔCRD), with the PRD deleted (Ror2ΔPRD) and with the intracellular domain deleted (Ror2ΔC) (Fig. 6A). In WT embryos at 72 hpf, the sy cartilage is a long stack of single cells, whereas in *ror2*^*−/−*^ animals, it is shorter, wider, and individual chondrocytes are often rounder than the more columnar WT cells (Fig. 6B,C). Expression of Ror2FL-sfGFP in chondrocytes rescued sy cartilage length in *ror2*^*−/−*^ animals (Fig. 6D,E). By contrast, Ror2ΔCRD-sfGFP failed to rescue, confirming that the Wnt-binding activity of the Ror2 CRD is required for stacking (Fig. 6F,G). Surprisingly, kinase-dead Ror2KD-sfGFP rescued sy cartilage length, indicating that Ror2 intrinsic kinase activity is dispensable for cartilage stacking (Fig. 6H,I). In contrast, expression of either Ror2ΔPRD-sfGFP or Ror2ΔC-sfGFP did not rescue sy cartilage stacking (Fig. 6J-M). We found that there was no statistical difference between the effectiveness of the Ror2FL and Ror2KD constructs in rescuing length (Fig. 6P). No significant difference was observed between Ror2ΔPRD or Ror2ΔC, demonstrating that the PRD specifically, but not the ST domains, is required for chondrocyte stacking (Fig. 6P). Lastly, we tested whether ectopic expression of a full-length Ror1-sfGFP construct (Ror1FL; Fig. 6A) was able to rescue symplectic cartilage stacking and found that in many instances it could (Fig. 6N-O,P). This result is consistent with partially redundant functions of Ror1 and Ror2 receptors in craniofacial cartilages. Together, these data suggest that Wnt5b binding to Ror2 and signaling and/or interactions via the PRD, but not kinase activity, are essential to transducing a cell polarity signal to shape cartilages of the zebrafish craniofacial skeleton.

## DISCUSSION

In this study we have shown requirements for the non-canonical Wnt receptors, Ror1 and Ror2, in cartilage morphogenesis in zebrafish. Like *wnt5b* mutants (Sisson et al., 2015), *ror1^−/−^; ror2^−/−^* double mutants display similarly severe craniofacial defects consistent with a loss of chondrocyte polarity, supporting the hypothesis that Wnt5b is the primary ligand for Ror1/2 receptors in cartilage. Our domain analysis of zebrafish Ror2 reveals that the extracellular Wnt-binding CRD and intracellular C-terminal portion of the receptor, specifically the PRD domain, are required for cartilage stacking. These data, taken together with the lack of a phenotype in *ror1^−/−^* mutants and increased severity of *ror1^−/−^; ror2^−/−^* double mutants compared with *ror2^−/−^* mutants, suggest that Wnt5b primarily binds Ror2 to mediate stacking, potentially by regulating cell adhesion and the cytoskeleton via the Ror2 PRD domain, whereas Ror1 plays a secondary role.

### A kinase-independent function of Ror2 in zebrafish cartilage morphogenesis

Ror1 is a pseudokinase, having no reported or predicted intrinsic kinase activity, and some evidence suggests that Ror2 similarly lacks kinase activity (Bainbridge et al., 2014; Katso et al., 1999; Mendrola et al., 2013; Sheetz et al., 2020). In order to test the role of the Ror2 putative kinase domain *in vivo*, we attempted to rescue cartilage stacking defects in zebrafish through the mosaic expression of a predicted kinase-dead Ror2 variant. The K507R mutation in human ROR2 targets a highly conserved residue in the kinase domain of RTKs that prevents ATP binding and abolishes the kinase activity of an RTK receptor *in vitro* (Matsuda et al., 2003). Human and zebrafish Ror2 are 72% identical and their kinase domains share 84% identity (Fig. S6). We found that a Ror2 mutant rescue construct with a mutation equivalent to K507R (K509R; Fig. S6), which is predicted to lack kinase activity, rescued symplectic cartilage length, demonstrating that this residue is dispensable for cartilage stacking. These results are consistent with Ror2 being a pseudokinase, or possibly that its kinase activity is not required specifically for cartilage morphogenesis. Even without the ability to phosphorylate targets directly, Ror2 may mediate phosphorylation of proteins indirectly through association with other kinases via its pseudokinase domain (Mendrola et al., 2013). Of note, small molecule inhibitors that can bind the non-functional ATP-binding sites within the pseudokinase domain may still be effective at downregulating signaling, suggesting that even without conferring kinase activity, these sites are important for downstream signaling (Sheetz et al., 2020).

### Conserved partially redundant functions for Ror1 and Ror2 in craniofacial development

*ror1^−/−^* mutant zebrafish have no apparent skeletal defects and appear to be phenotypically normal. *Ror1* hypomorphic mutant mice are smaller than their littermates and die shortly after birth. Though initially reported to have no skeletal abnormalities (Nomi et al., 2001), mutants have subtle defects in the axial skeleton (Lyashenko et al., 2010), though *Ror1^−/−^* mice have apparently normal appendicular skeletons (Ho et al., 2012). Both *Ror1* and *Ror2* are expressed throughout the developing mouse embryo, including in the first and second pharyngeal arches and developing limbs, with largely overlapping patterns (Al-Shawi et al., 2001; Matsuda et al., 2001). Both receptors can also form heterodimers in certain contexts (Paganoni et al., 2010). Zebrafish *ror1* and *ror2* are expressed ubiquitously throughout gastrulation but expression becomes more restricted to the head between 24 and 60 hpf (Bai et al., 2019; Bai et al., 2014). We detected *ror1* mRNA in mesenchyme closely associated with stacking cartilages in the zebrafish head, with enrichment at the site of the Mc–pq joint region, though we observe no specific defects in the jaw joint in *ror1* mutants. We cannot exclude the possibility that low levels of *ror1* are present in cartilages, as we do observe some punctate signal in chondrocytes. *Ror1/2* double knockout mice have more severe skeletal phenotypes than in loss of *Ror2* alone and are similar in severity to *Wnt5a* knockout mice (Ho et al., 2012). Consistent with these observations, we found that *ror1^−/−^; ror2^−/−^* double mutants had more severe craniofacial defects than *ror2* mutants and resembled those of *wnt5b* mutants but that *ror2^−/−^; wnt5b^−/−^*double mutants were phenotypically similar to *wnt5b^−/−^.* In addition, our *ror2^−/−^* symplectic cartilage stacking rescue experiments employing a Ror1-sfGFP construct showed that Ror1 can compensate for the loss of Ror2 in some situations. Therefore, it is likely that Ror1 and Ror2 function partially redundantly in zebrafish craniofacial cartilages. *Ror1* and *Ror2* also genetically interact with *Wnt9a*, enhancing phenotypes in the limb and palate (Weissenbock et al., 2019) whereas, on its own, *Wnt9a* mutants display only a modest shortening of the limbs (Spater et al., 2006). Zebrafish *wnt9a* mutants have a mildly shorter palate than WT animals (Rochard et al., 2016) but no other skeletal phenotypes have been reported.

### Potential cytoskeletal targets of Wnt5b-activated Ror2 and roles in focal adhesion formation in cartilage precursors

Our data suggest that Ror2-mediated cartilage morphogenesis requires: (1) Wnt5b binding to the Ror2 CRD, and (2) the Ror2 intracellular PRD. This is consistent with previous work indicating that Wnt5b, but not Wnt5a, plays a primary role in Wnt-PCP signaling during zebrafish skeletal morphogenesis (Sisson et al., 2015). Mammalian Wnt5b also binds Ror2 and regulates chondrocyte progenitor migration (Bradley and Drissi, 2011).

What are the downstream effectors of Wnt5b-activated Ror2? Cartilage morphogenesis requires polarized cell-cell intercalation, a process that relies on directed cytoskeletal dynamics and adhesion. We show that the zebrafish Ror2 PRD is required for its function in cartilage morphogenesis. In cell culture, the Ror2 PRD can interact with the actin-binding protein Filamin A (FLNA), providing a direct physical link between the Ror2 receptor and the cytoskeleton (Nishita et al., 2006). In addition, FlnA is required for Wnt5a-induced and Ror2-dependent polarized cell migration during wound-healing (Nomachi et al., 2008). Though *flna* is expressed throughout the head at 24 hpf, expression at later stages, such as during craniofacial cartilage morphogenesis, has not been determined (Liang et al., 2020). Future studies will have to determine whether Ror2 physically interacts with Flna in the context of cartilage morphogenesis.

Consistent with a role in adhesion, we show reductions in the number of pFAK foci at the cell membranes of chondrocyte precursors in Ror2 mutants. FAs link the ECM to the actin cytoskeleton through Itgs and have important roles in cell migration, mechanosensation and cell signaling. Itgs (Aszodi et al., 2003; Bengtsson et al., 2005) and integrin-linked kinase (ILK) (Grashoff et al., 2003) regulate chondrocyte polarity, shape and growth plate patterning, and their loss can cause chondrodysplasias. FAK phosphorylation is associated with the formation of FAs, Itg signaling and the regulation of downstream signaling, including Rho GTPases as well as actin remodeling (Kleinschmidt and Schlaepfer, 2017; Mitra et al., 2005). Reduction or loss of FAs likely results in downregulated Itg-based signaling and weakened chondrocyte-ECM adhesion, which is consistent with the rounder chondrocyte aspect observed in Ror2-deficient zebrafish. What is the mechanistic relationship between Ror2 and FA formation? WNT5A-bound ROR2 activates JNK, a direct activator of FAK, in wound-healing assays in a FLNA-dependent manner (Nomachi et al., 2008); however, additional studies are required to directly test whether the ROR2 PRD might mediate FAK phosphorylation through FLNA. Alternatively, the reduction in FAs in *ror2* mutants might be a secondary consequence of a more general loss of cell polarity. Experiments that address this possibility are necessary to establish a mechanistic link between FAs and Ror2 signaling.

### Regulation of skeletal morphogenesis by Ror2 and PCP

Growing evidence suggests that Ror2 plays an essential role in non-canonical Wnt signaling, both in skeletal development and evolution. Human mutations in ROR2 can cause Robinow syndrome (Afzal et al., 2000; Lima et al., 2022; Person et al., 2010; van Bokhoven et al., 2000; White et al., 2018), as well as Brachydactyly type B1 (Oldridge et al., 2000; Schwabe et al., 2000), including autosomal recessive forms resembling our *ror2^−/−^* mutant zebrafish. Both of these disorders are characterized by defects in elongation of endochondral bones, consistent with defects in cartilage morphogenesis during embryogenesis and later in developing growth zones. Mutations in Ror2 have also recently been linked to diversity in craniofacial morphologies in domesticated pigeons (Boer et al., 2021), including beak length, which also depends on maxillary/mandibular skeletal elongation.

Our data suggest that the common processes disrupted by loss of Ror2 in these contexts are the polarized cell-cell intercalations that drive cartilage morphogenesis, which require the non-canonical Wnt-PCP pathway. Wnt5 orthologs and Ror2 form a small group of PCP factors that regulate both cartilage morphogenesis and CE of the axial mesoderm during vertebrate gastrulation, specifically Wnt5a in tetrapods and Wnt5b in zebrafish (Andre et al., 2015; Bai et al., 2014; Schambony and Wedlich, 2007). Wnt-PCP signaling also involves Wnt11 and Vangl2. In zebrafish, *wnt11^−/−^* (now known as *wnt11f2*^−/−^) and *vangl2^−/−^* mutants have strong CE defects (Heisenberg et al., 2000; Solnica-Krezel et al., 1996) but few, if any, defects in cartilage (Sisson et al., 2015). These data suggest that mechanisms controlling cell polarity in CE in zebrafish only partially overlap with those that drive cartilage morphogenesis later during embryonic development. We have previously shown that a second PCP pathway, involving the atypical protocadherins Fat3a and Dchs2, is required for shaping embryonic cartilages (Le Pabic et al., 2014). Future studies are needed to determine whether the non-canonical Wnt and Fat-Dachsous pathways interact during cartilage morphogenesis.

## MATERIALS AND METHODS

### Fish lines

Wild-type AB fish were crossed to *Tg(sox10:lyn-tdTomato) ir1040* animals and their progeny were co-injected at the one-cell stage with gRNA and Cas9 RNA to produce *ror1* and *ror2* mutants. Alleles recovered were *ror1* (+4 bp insertion) *ir1095*, *ror1* (−4 bp deletion) *ir1096*, *ror2* (−1 bp deletion) *ir1093*, and *ror2* (−4 bp deletion) *ir1094*. *ror1 ir1095* and *ror2 ir1093* were used to generate the data presented here. *wnt5b*(*pipetail*)*^ta98^* mutants were used for Alcian Blue-Alizarin Red staining*.* All embryos and fish were maintained under standard conditions (Westerfield, 2000) in accordance with University of California, Irvine, Institutional Animal Care and Use Committee protocols.

### CRISPR-Cas9 mutagenesis

gRNAs were designed using CHOPCHOP (Labun et al., 2019). Template-based assembly of gRNA oligos was performed using the 5′ primer sequence GCAGCTAATACGACTCACTATAG[target sequence]GTTTTAGAGCTAGAAATA and the universal 3′ primer sequence AAAAGCACCGACTCGGTGCCACTTTTTCAAGTTGATAACGGACTAGCCTTATTTTAACTTGCTATTTCTAGCTCTAAAAC. The *ror1* 5′ primer sequence was: GCAGCTAATACGACTCACTATAGgatggagtccccgaaacgGTTTTAGAGCTAGAAATA (gRNA target in lowercase). The *ror2* gRNA 5′ primer sequence was as follows: GCAGCTAATACGACTCACTATAGgcgtcgttctt cagccaaGTTTTAGAGCTAGAAATA (gRNA target in lowercase). gRNA template DNAs were used in reverse transcription reactions (MEGAshortscript T7 transcription kit, AM1354, Invitrogen) to produce gRNAs. Zebrafish codon-optimized nCas9n mRNA was *in vitro* transcribed from XbaI-linearized pT3TS-nCas9n plasmid DNA (Addgene plasmid #46757; Jao et al., 2013) using the mMessage mMachine T3 kit (Invitrogen, AM1348). One-cell stage embryos were co-injected with *ncas9n* RNA and gRNA, then raised to adulthood. Sperm from adult males was sampled and used as a template in PCR reactions with genotyping primers *ror1_fwd* TCTGGCTTGTCTTTTCAGATCA and *ror1_rev* ACACTGAAAGTAGCCGGTGTCT or *ror2_fwd* GGAGTTTCTGGAGCAGCCAA and *ror2_rev* AGTCGCACATACAGCACTCC. Heteroduplex mobility assays (HMA) were performed with PCR products on 10% native PAGE gels and males harboring germline mutations were isolated according to their unique band patterns (Ota et al., 2013). PCR products were then TA-cloned using the pGEM-T Easy kit (Promega, A1360) and sequenced. F1s were intercrossed and F2s were identified as heterozygous by their unique PAGE gel heteroduplex band pattern or as homozygous WT or mutant by sequencing of PCR products that appeared as homoduplexes by native PAGE.

### Cartilage and bone stains

Acid-free Alcian Blue-Alizarin Red double stains were performed on 5 dpf fish as described (Walker and Kimmel, 2007). Cartilages were dissected, flat-mounted in 80% glycerol and imaged on a Zeiss AxioPlan 2 Imaging microscope using either a MicroPublisher 5 RTV camera (QImaging) with Volocity software (Quorum Technologies) or a Zeiss AxioCam 305 color camera with Zeiss ZEN Blue software. For particularly thick cartilages (e.g. *wnt5b^−/−^* mutants), focus stacking/focal plane merging was manually performed on a *z*-series of images to produce single images where most of the cartilages are in focus.

### *In situ* hybridization

RNA *in situ* hybridization was performed as previously described (Thisse and Thisse, 2008). Probes used were *sox9a* (Chiang et al., 2001), *ror1*, *ror2*, *wnt5b* (Rauch et al., 1997) and *gpc4.* The *ror1* probe template encompassing the last exon and 3′ untranslated region was PCR amplified from WT genomic DNA using the following primers: fwd GCCACCAGAGGCCATAGTTT and rev GGATCCTAATACGACTCACTATAGGGTAAGGCCGTTCTGCCACATT (T7 promoter sequence underlined). The *ror2* probe plasmid was produced by PCR amplification of an 807 bp fragment from 54 hpf WT cDNA (using primers: fwd ACAGATGCAGGGAGAAAGTG and rev TTTGCCTGATGTTTGTGCTG) and then TA-cloned into pCR4-TOPO (Invitrogen, K457502). The *gpc4* probe plasmid was produced by PCR amplification of a 564 bp fragment from 54 hpf WT cDNA (using primers: fwd TTGTGCGGACGTATGGCTTG and rev CCAGACAGCCCCTCATGACA) and then TA-cloned into pCRII-TOPO (Invitrogen, K457501). DIG-labeled anti-sense RNA probes were transcribed from linearized plasmid DNA (*sox9a*, linearized with EcoRV; *ror2*, linearized with NotI; *gpc4*, linearized with BamHI; *wnt5b*, linearized with KpnI) or purified PCR product using T3 (for *ror2*), T7 (for *sox9a*, *ror1*, *gpc4*) or SP6 (for *wnt5b*) RNA polymerase (Roche, 11031163001,10881767001 or 10810274001, respectively) and DIG RNA labeling Mix (Roche, 11277073910). HCR probes for *ror1* (NCBI reference sequence XM_005165885.4, 20 probe set in B3) and *ror2* (NCBI reference sequence XM_021472213.1, 20 probe set in B2) were designed and produced by Molecular Instruments. Amplifiers used were B3 Alexa Fluor 647 and B2 Alexa Fluor 488 (Molecular Instruments). HCR RNA-FISH was performed as described in the ‘whole-mount zebrafish embryos and larvae’ protocol available on the Molecular Instruments website (https://www.molecularinstruments.com/hcr-rnafish-protocols) and all embryos were stained with 10 mg/ml DAPI at 1:1000. Traditional *in situ* hybridizations were imaged on a Zeiss AxioPlan 2 imaging microscope using either a MicroPublisher 5 RTV camera (QImaging) with Volocity software (Quorum Technologies) or a Zeiss AxioCam 305 color camera with Zeiss ZEN Blue software. HCR *in situ* hybridizations were imaged using an SP8 confocal microscope (Leica).

### Immunohistochemistry

Embryos were fixed in 4% paraformaldehyde for 2 h at room temperature or overnight at 4°C. After fixation, embryos were permeabilized in ice-cold 100% acetone at −20°C for 7 min. After washing in PBS-DT (1× PBS, 0.5% Triton X-100, 1% DMSO), embryos were permeabilized again in PBS-DT (1% Triton X-100 for FAK or γ-tubulin staining) for 1 h. Rabbit anti-phospho-FAK (Tyr861) (Invitrogen, 44-626G) was used at 1:200, rabbit anti-γ-tubulin (GeneTex, GTX113286) at 1:250, and mouse anti-Ror2 (Developmental Studies Hybridoma Bank, AB_10804796) at 1:100. Donkey anti-rabbit Alexa Fluor 647 (Jackson ImmunoResearch, 711-606-152) and donkey anti-mouse Alexa Fluor 488 (Jackson ImmunoResearch, 715-546-150) secondaries were used at 1:500. Alexa Fluor 647 Phalloidin (Thermo Fisher Scientific, A22287) was used at 1:50. All embryos were stained with 10 mg/ml DAPI at 1:1000. Primary and secondary antibody incubations were performed overnight at 4°C. All fluorescent imaging was performed on an SP8 confocal microscope.

### Imaging of adult zebrafish

Adult zebrafish were anesthetized in tricaine and immobilized on a bed of 3 or 4% methylcellulose in a glass dish, which was then filled with tricaine in system water. Fish were imaged using a Zeiss Stemi-2000 stereomicroscope with a Zeiss AxioCam HRc color camera and ZEN Blue software for image acquisition.

### Transgenic rescue constructs

RNA was extracted from 2 dpf zebrafish with TRIzol Reagent (Invitrogen, 15596026) and used in a reverse transcription reaction to produce cDNA (Proscript II First Strand cDNA Synthesis Kit, New England Biolabs, E6560L). Zebrafish Ror1 and Ror2 constructs were amplified from 2 dpf cDNA and sfGFP fusion constructs were produced using Gibson Assembly (Gibson et al., 2009) with NcoI/XbaI-digested Tol2Kit plasmid #455 (pME-EGFP no stop) serving as the vector backbone (Kwan et al., 2007). Ror2KD was produced by introducing the K509R mutation, equivalent to the mammalian kinase-dead K507R mutation (Fig. S4). LR reactions were performed with p5E-sox10 (-4.8) or p5E-col2a1a (-1.8) (Dale and Topczewski, 2011) and Tol2kit plasmids p3E-polyA (#302) and pDestTol2pA2 (#394) or pDestTol2CG2 (#395), and the resulting transgenes were co-injected with Tol2 mRNA into one-cell stage *ror2^−/−^* embryos as previously described (Kwan et al., 2007).

### pFAK quantifications

A *z*-stack encompassing the whole sy cartilage from anti-pFAK(Y861)-stained *sox10:lyn-tdTomato* transgenic WT or *ror2^−/−^* animals was acquired and then analyzed using ImageJ. Brightness/contrast was adjusted to remove some background signal and facilitate easier counting (image>adjust>brightness/contrast). For both the anti-pFAK(Y861) and *sox10:lyn-tdTomato* channels, the minimum displayed value was set to 20 and the maximum displayed value was set to 100. The multi-point tool was used to label and track pFAK foci through each *z*-stack.

### Cartilage measurements

All cartilage and tr cell measurements were performed using ImageJ. Tr cartilage cells were individually traced with the freehand selection tool. Next, ‘shape descriptors’ was selected under ‘analyze>set measurements’ to calculate the aspect ratio (AR) of the selected tr cell. Sy cartilages differ in width along their length, so we averaged together three widths along the length (as illustrated in Fig. 3M) to generate a ‘width’ value. Sy cartilage lengths were determined by drawing and measuring a segmented line through the middle of the cartilage from posterior to anterior, as illustrated in Fig. 3M. Pq cartilages (minus the pterygoid process) were traced with the freehand or polygon selection tool. An ellipse was fitted to the selection by selecting ‘edit>selection>fit ellipse’ as depicted in Fig. 3N. Next, ‘shape descriptors’ was selected, then the selection measured to determine the AR for each pq cartilage. For ch cartilages, a line we defined as the length was drawn along the long-axis of the ch cartilage and measured. A line we defined as the width was drawn along the short-axis at the midpoint of the ch cartilage and measured (as illustrated in Fig. 3O). A diagram illustrating the measured lengths for the anterior neurocranium (An) can be found in Fig. 3P. Briefly, a line we defined as the length was drawn along the long-axis of the An and measured. A line we defined as the width was drawn between the two points furthest away from each other along the short-axis of the An, then measured.

### Symplectic cartilage polarity quantifications

A *z*-stack encompassing the entirety of an sy cartilage from anti-γ-tubulin-stained *sox10:lyn-tdTomato* transgenic WT or *ror2^−/−^* animals was acquired and then analyzed in ImageJ. The multi-point tool was used to label and track MTOCs through each *z*-stack. Labelled MTOCs were binned into one of four quadrants as depicted in Fig. 4M.

### Ror2 kinase domain sequence alignment and residue identity

Protein sequence alignment was performed using the Clustal Omega (1.2.4) multiple sequence alignment tool (McWilliam et al., 2013). Zebrafish (NCBI reference sequence: XP_689681.6) and human (NCBI reference sequence: NP_004551.2) Ror2 protein sequences were used as input. Percent identity was determined by Clustal 2.1 as an output of Clustal Omega.

### Plots and statistical analyses

All plots were produced and statistical analyses performed using RStudio. Packages used were ggplot2, RColorBrewer, rstatix and Circular. Statistical tests used were the Wilcoxon rank-sum test (for embryo length, tr cell Major Axis/Minor axis and pY861 foci comparisons); Kruskal-Wallis test with post-hoc Dunn’s test and Bonferroni correction (for cartilage Length/Width and Major Axis/Minor Axis comparisons, and Ror2 domain analysis); and Watson’s two-sample test for homogeneity (for angular polarity data).

## Supplementary Material

10.1242/develop.201273_sup1 Supplementary information
