# Supplementary material for: The non-canonical Wnt receptor Ror2 is required for cartilage cell polarity and morphogenesis of the craniofacial skeleton in zebrafish
Source: Development. 2023 Apr 21;150(8):dev201273. doi: 10.1242/dev.201273 (PMC10163346; doi:10.1242/dev.201273)
Supplement: Supplementary information [file develop-150-201273-s1.pdf]

Figure S1

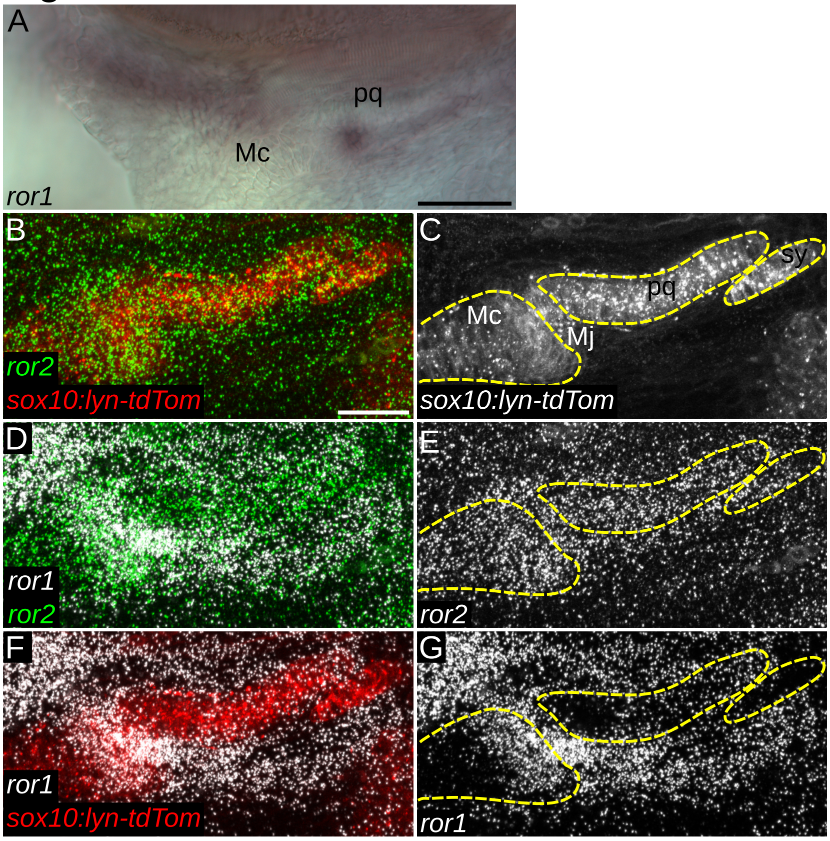

Fig. S1. *ror1* and *ror2* are expressed at the Meckel's joint and in chondrocytes at 72 hpf.

(A) In situ hybridization for ***ror1*** in a 72 hpf wild-type (WT) embryo. (B-G) HCR for ***ror1*** and ***ror2*** in a 72 hpf WT ***Tg(sox10-lyn-tdTomato)*** ("***sox10:lyn-tdTom***") embryo. ***ror1*** in white, ***ror2*** in green, and ***sox10:lyn-tdTom*** in red (B, D, F). Grayscale (C, E, G). Panels B-G are z-projections. Mc, Meckel's cartilage; pq, palatoquadrate cartilage; sy, symplectic cartilage; ch, ceratohyal cartilage; Mj, Meckel's joint. Mc, pq, and sy cartilages are outlined in dotted yellow line. Anterior to the left in all panels. Scale bars = 25 µm for A-I.

Development • Supplementary information

Figure S2

A

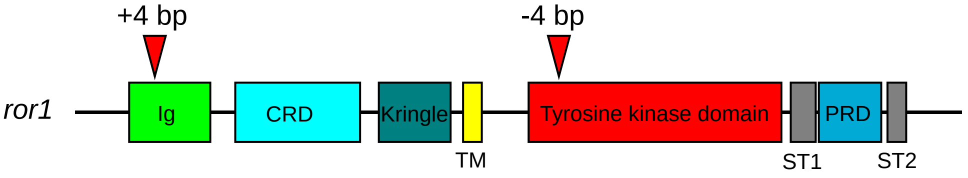

DNA sequences

ror1 WT (gRNA1): GGACAGACAGCTGAGTTGCATTGCC**ACGTTTCGGGGACTCCATC**ACCCTCAGTCCGCTGGCTT

ror1 (+4 bp)^{ir1095}: GGACAGACAGCTGAGTTGCATTGCC**ACGTtgcaTTCGGGGACTC**CATCACCCTCAGTCCGCTGGCTT

ror1 WT (gRNA2): AGTAAAGCCAAGGAGCTTCCT**CTGTCTGCGGTACGCTTC**ATGGAGGAGCTGGGCGAGAGTGCCTT

ror1 (-4 bp)^{ir1096}: AGTAAAGCCAAGGAGCTTCCT**CT----GCGGTACGCTTC**ATGGAGGAGCTGGGCGAGAGTGCCTT

Protein sequences

Ror1 WT:
MYERLRAEEMSRGTVKEPHGCQRNFTLICVLLALSCGAEIPSDPSVVSSSSWNMSRAAERDHFLRLDAPMNNITTSLGQTAELHCHVSGTPSPSVR
WLKNDAPVVQEPRRVSYRPTPYGSRLRIKNLDTTDTGYFQCVATNSYGTVSTTGILFVKFDPAPTPLPGRPSPEDFDEEGFCQPYRGIACARFIGN
RSIFVDSLQMQGEIETQITAAFTMIGTSNHLSDRCSQFATVPSLCHFAFPTCDRSSGMDKPRDLCKDECEILENDLCKTEYIIARSNPLILKRLKL
PKCEDLATLDSPEAANCMRIGIPIAETINKSHKCYNNSGSDYRGTVSVTKCPGRQCQPWNSQYPHSHTFLAMRYPELNGGHSYCRNPGNKHDAPWC
FTLDEAVRMELCDIPPCDLPKHGSSSNSMGILYILVPSVAIPLAIALLFFFICVCRNNQKASRPPAPRQPKPVRGQNVEMSMLTAYKPKSKAKELP
LSAVRFMEELGESAFGKIYKGHLYLPGMEHAQLVAIKTLKDISSAQHWGDFQQEASVLAELHHPNVVCLLGVVTQEQPVCMLFEFLAQGDLHEFLI
MRSPHSDVGCSSDEDGTVKSSLDHGDFLHLAIQVAAGMEYLASHFFIHKDLAARNILVGEQLHIKISDLGLSREIYSSDYYRVQPNTLLPIRWMPP
EAIVYGKYSTDSDVWAFGVVLWEIFSFGLQPYYGFSNQEVMEMVRKRQLLPCPEDCPPRMYALMTECWQEGPARRPRFKDIHTRLRAWEGLSSHAS
SSTPSGGNATTQTTSLSASPVSNLSSPRYAGYIYGAPQSLQPGQITGFMAAPMPQSQRFIPVNGYPIPTGYAAFPAAHFAASQGPPQVVQHCPPPK
SRSPSSASGSTSTGHVTSVPSTGSNHDANTPLLSHCVTLTPMTAVPGGTLPVFGHMAQKAMQIDPSQAALLADTNRLMYSESIITADL*

Ror1^{ir1095} (+4 bp):
MYERLRAEEMSRGTVKEPHGCQRNFTLICVLLALSCGAEIPSDPSVVSSSSWNMSRAAERDHFLRLDAPMNNITTSLGQTAELHCHVAFGDSITLS
PLA*

Ror1^{ir1096} (-4 bp):
MYERLRAEEMSRGTVKEPHGCQRNFTLICVLLALSCGAEIPSDPSVVSSSSWNMSRAAERDHFLRLDAPMNNITTSLGQTAELHCHVSGTPSPSVR
WLKNDAPVVQEPRRVSYRPTPYGSRLRIKNLDTTDTGYFQCVATNSYGTVSTTGILFVKFDPAPTPLPGRPSPEDFDEEGFCQPYRGIACARFIGN
RSIFVDSLQMQGEIETQITAAFTMIGTSNHLSDRCSQFATVPSLCHFAFPTCDRSSGMDKPRDLCKDECEILENDLCKTEYIIARSNPLILKRLKL
PKCEDLATLDSPEAANCMRIGIPIAETINKSHKCYNNSGSDYRGTVSVTKCPGRQCQPWNSQYPHSHTFLAMRYPELNGGHSYCRNPGNKHDAPWC
FTLDEAVRMELCDIPPCDLPKHGSSSNSMGILYILVPSVAIPLAIALLFFFICVCRNNQKASRPPAPRQPKPVRGQNVEMSMLTAYKPKSKAKELP
LRYASWRSWARVPSVRSTKATCTCLAWSTLNLWP*

B

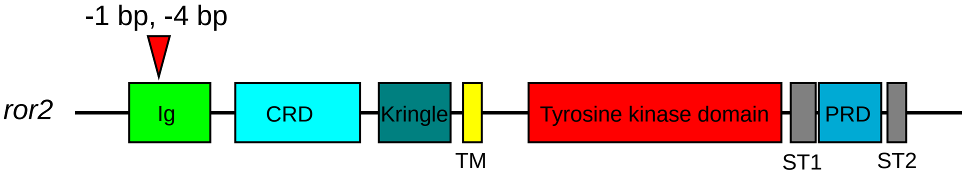

DNA sequences

ror2 WT: CAGGACACCCAAGGCCCAGTATCCG**TTGGCTGAAGAACGACGC**TCCTGTGGTGCAAGAACAGGGCC

ror2 (-1 bp)^{ir1093}: CAGGACACCCAAGGCCCAGTATCCG**Tc-GCTGAAGAACGACGC**TCCTGTGGTGCAAGAACAGGGCC

ror2 (-4 bp)^{ir1094}: CAGGACACCCAAGGCCCAGTATCCG**----CTGAAGAACGACGC**TCCTGTGGTGCAAGAACAGGGCC

Protein sequences

Ror2 WT:
MFPVRMYHILRITTGAHLVATFIFFATFAYAEADVNLFESEEIEALAEAQSGGLPTPEGYYLEFLEQPNNITIVQGQTATLHCKVAGHPRPSIRWL
KNDAPVVQEQGRVSIRKTEAGSKLRIQDLDTTDTGYYQCVATNSIKVISATGVLYVRLGNTGQSPAHGAEDSTHEKGFCQPYRGIACARFIGNRSI
YVESLQMQGESENRITAAFTMIGTSTQLSDQCSEFAIPSFCYYVFPLCEEGTRGPRRRQLCRDECEALENDLCNAEYTIARSNPLILMQLELPVCS
LLPHPGSPDASSCIRIGLPPQYSPPDHSCYNESGGNYKGTVSVTKSGFQCQPWSAQYPHSHHLSPIEYPQLRGGHNFCRNPGGQMESPWCFTLDPN
VRVDLCDIPPCNPPEKMKKELLYILIPSVATPLVFACLFFLICMCRNKQKESVDTPTRRQLTASPSQEMELPLLNQHKHQAKLRELNLSAVRFMEE
LGEDRFGKVYKGHLYGTAPGEQTQVVAIKTVKDKDEGPLREEFRHEAMLRSRLQHPNIVCLLGVITKEQPMSMIFSYSGQGDLHEFLVMRSPHSDV
GSSDDDKTVKSTLEQADFLHIVTQVAAGMEYLSSHHVVHKDLAARNILVCDKLNVKILDLGLFREVYSADYYKLMGASPFPIRWMSPEAILYGKFS
TDSDIWSYGVVLWEIFSYGLQPYCGYSNQDVIEMVRNRQVLSCPDDCPAWIYTMMLECWNEFPSRRPRFKDIHARLRTWESLSNYNSSAQTSGASN
TTQTSSLSTSPVSNISNARYVSPKKSSPFPQPQFVSMKGQMRPMVPPQLYIPVNGYQPMPAYPYLQNFYPMQIPMQMPPQQMHPQMVAKAGSHHSG
SGSTSTGYVTTAPSNGSGTDKVALMNEDLKATEDDFADGASQKGLHHEDLSVPETELLGDNDSPHTEDNEINSDA*

Ror2 (-1 bp)^{ir1093}:
MFPVRMYHILRITTGAHLVATFIFFATFAYAEADVNLFESEEIEALAEAQSGGLPTPEGYYLEFLEQPNNITIVQGQTATLHCKVAGHPRPSIRR*

Ror2 (-4 bp)^{ir1094}:
MFPVRMYHILRITTGAHLVATFIFFATFAYAEADVNLFESEEIEALAEAQSGGLPTPEGYYLEFLEQPNNITIVQGQTATLHCKVAGHPRPSIR*

Fig. S2. ror1 and ror2 gRNA target sites.
(A, B) Diagrams of zebrafish *ror1* (A) and *ror2* (B) genes with domains annotated. Red triangles indicate gRNA target sites. Ig, immunoglobulin-like domain; CRD, cysteine-rich domain; Kringle, Kringle domain; TM, transmembrane domain; ST1, serine-threonine domain 1; ST2, serine-threonine domain 2; PRD, proline-rich domain.

Development • Supplementary information

Figure S3

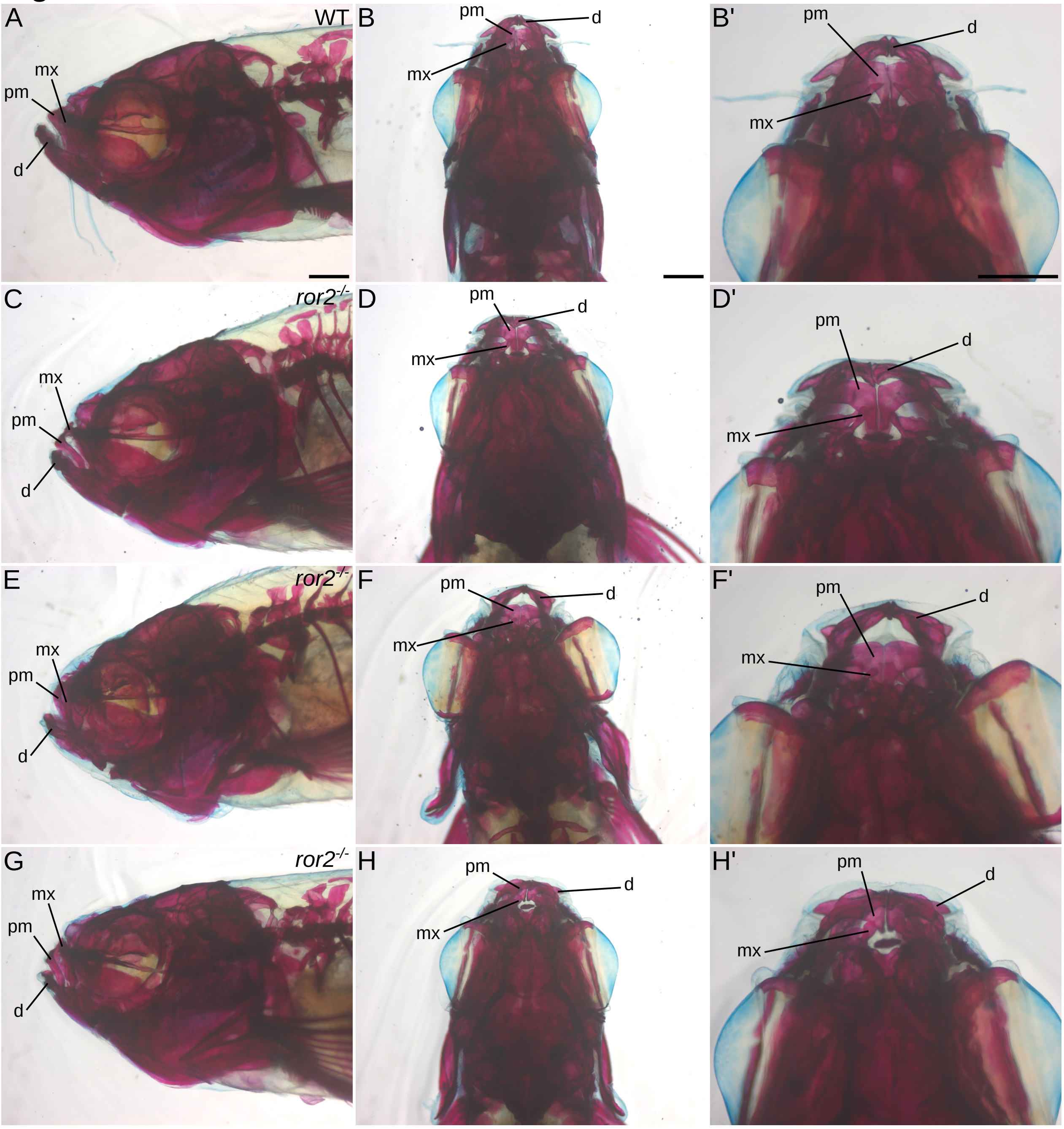

Fig. S3. Dysmorphic craniofacial skeletons of adult ror2 mutants.
(A-H') Alizarin red stained adult head skeletons. A representative wild type (WT; A-B') and 3 examples of *ror2*$^{-/-}$ animals (C-H'). A, C, E, and G are lateral views. B, D, F, and H are dorsal views. (B', D', F', and H') Magnified views of B, D, F, and H, respectively. d, dentary; pm, premaxilla; mx, maxilla. Scale bars = 1 mm for all panels.

Figure S4

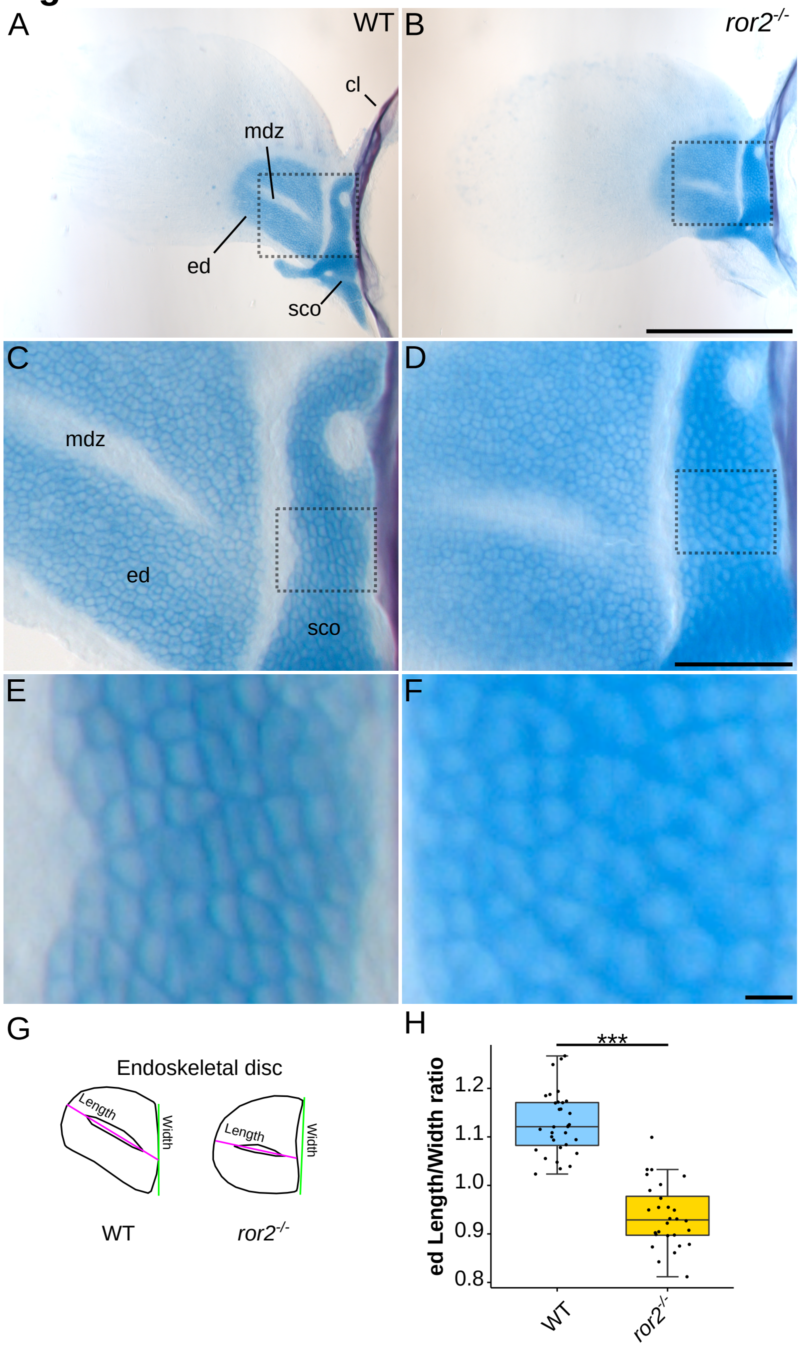

Fig. S4. Abnormal pectoral fin development in *ror2* mutants. Representative wild type (WT; A) and *ror2*$^{-/-}$ (B) pectoral fins at 30 dpf. Panels C and D are magnified views of the boxed regions in A and B, respectively. Panels E and F are magnified views of the boxed regions in C and D, respectively. Diagrams of representative endoskeletal discs of 0.70 mm eye diameter WT and *ror2*$^{-/-}$ mutants indicating measured features (G). (H) Quantification of endoskeletal disc length-to-width ratios. Standard length (SL) = 7.80 mm, eye diameter = 0.70 mm for WT in A, C, and E, and SL = 6.85, eye diameter = 0.70 mm for *ror2*$^{-/-}$ in B, D, and F. n=32 endoskeletal discs from 16 0.70 mm eye diameter WT and 28 endoskeletal discs from 14 0.70 mm eye diameter *ror2*$^{-/-}$ animals for H. Eye diameter is used to normalize between genotypes as *ror2* mutants are shorter than WT. Wilcoxon rank-sum test, ***p<0.001 for H. Cl, cleithrum; ed, endoskeletal disc; mdz, zone of matrix decomposition; sco, scapulocoracoid. Scale bar = 0.5 mm for A and B, 100 µm for C and D, 10 µm for E and F.

Figure S5

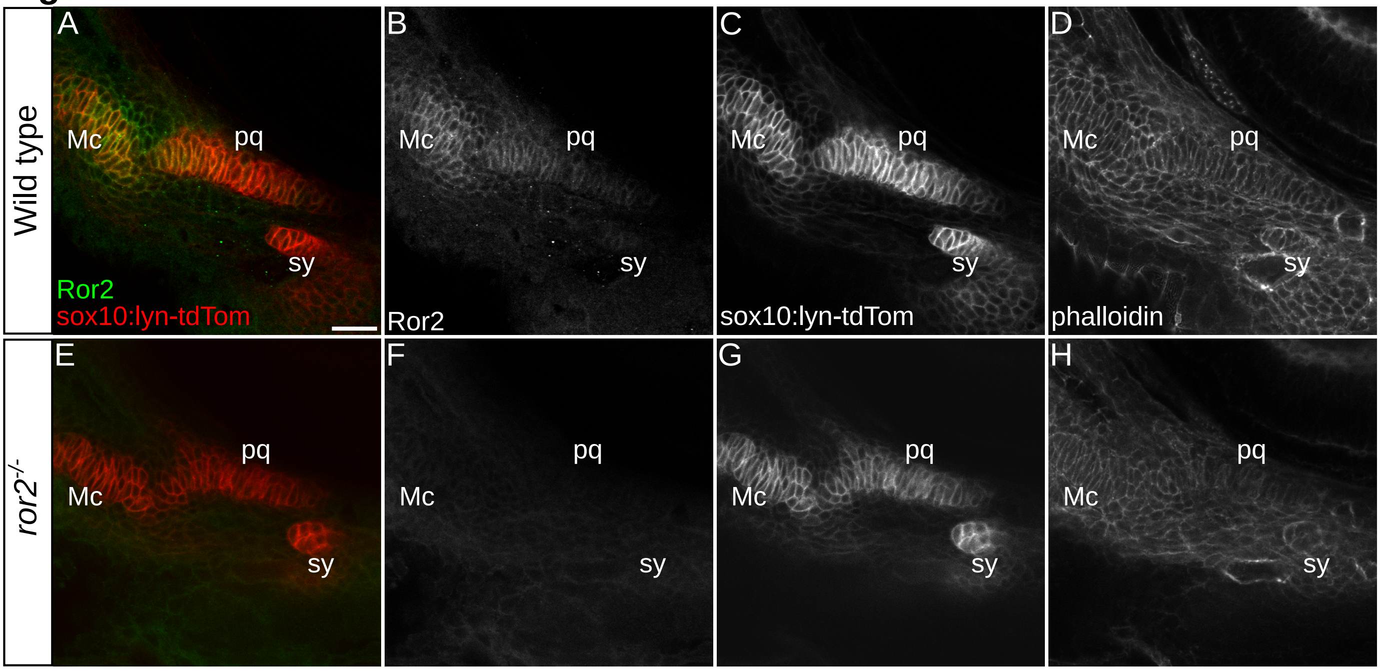

Fig. S5. Ror2 protein is expressed in cartilage progenitors.

(A-H) Anti-Ror2 antibody staining in 54 hpf wild type (WT) (A-D) and maternal-zygotic (*MZ-ror2*) *ror2*$^{-/-}$ mutants (E-H) embryos. Merged images of anti-Ror2 staining in green and *sox10:lyn-tdTomato* in red (A, D). Grayscale images of anti-Ror2 alone (B, F), *sox10:lyn-tdTomato* (C, G), and Alexa Fluor 647 Phalloidin (D, H). Mc, Meckel's cartilage; pq, palatoquadrate cartilage; sy, symplectic cartilage. Scale bar = 20 µm for A-H.

Figure S6

A

```
                                                    K509
zfish_ror2    475        vrfmeelgedrfgkvykghlygtapgeqtqvvaiktvkdkdegplreefrheaml     529
human_ror2    473        vrfmeelgedrfgkvykghlfgpapgeqtqavaiktlkdkaegplreefrheaml     527
                         ********************:* *******.*****:*** **************
                                                    K507

zfish_ror2    530   rsrlqhpnivcllgvitkeqpmsmifsysgqgdlheflvmrsphsdvgssdddktvkstl     589
human_ror2    528   rarlqhpnvvcllgvvtkdqplsmifsycshgdlheflvmrsphsdvgstdddrtvksal     587
                    *:******:******:**:**:******..:*****************:***:****:*

zfish_ror2    590   eqadflhivtqvaagmeylsshhvvhkdlaarnilvcdklnvkildlglfrevysadyyk     649
human_ror2    588   eppdfvhlvaqiaagmeylsshhvvhkdlatrnvlvydklnvkisdlglfrevyaadyyk     647
                    *  **:*:*:*:*****************:**:** ******* *********:*****

zfish_ror2    650   lmgaspfpirwmspeailygkfstdsdiwsygvvlweifsyglqpycgysnqdviemvrn     709
human_ror2    648   llgnsllpirwmapeaimygkfsidsdiwsygvvlwevfsyglqpycgysnqdvvemirn     707
                    *:* * :*****:****:***** *************:****************:**:**

zfish_ror2    710   rqvlscpddcpawiytmmlecwnefpsrrprfkdiharl                          748
human_ror2    708   rqvlpcpddcpawvyalmiecwnefpsrrprfkdihsrl                          746
                    **** ********:*::*:*****************:**
```

Fig. S6. Sequence alignment of human and zebrafish Ror2 kinase domains. Sequence alignment of zebrafish (“zfish_ror2”; amino acids 475-748) and human (“human_ror2”; amino acids 473-746) Ror2 kinase domains produced by Clustal Omega. Asterisks indicate identical residues; colons, strongly similar residues; and periods, weakly similar residues. Red text indicates small hydrophobic amino acids; blue, acidic amino acids; magenta, basic amino acids; green, amino acids with hydroxyl, sulfhydrl, or amine groups, or glycine. Blue highlight indicates the conserved lysine associated with kinase activity.

Development • Supplementary information
