# Supplementary material for: The non-canonical Wnt receptor Ror2 is required for cartilage cell polarity and morphogenesis of the craniofacial skeleton in zebrafish
Source: Development. 2023 Apr 21;150(8):dev201273. doi: 10.1242/dev.201273 (PMC10163346; doi:10.1242/dev.201273)
Supplement: Supplementary file 1 [file develop-150-201273_review_history.pdf]

The non-canonical Wnt receptor Ror2 is required for cartilage cell polarity and morphogenesis of the craniofacial skeleton in zebrafish

Daniel B. Dranow, Pierre Le Pabic and Thomas F. Schilling
DOI: 10.1242/dev.201273

Editor: Steve Wilson

Review timeline

Original submission:	2 September 2022
Editorial decision:	13 October 2022
First revision received:	15 February 2023
Editorial decision:	13 March 2023
Second revision received:	14 March 2023
Accepted:	21 March 2023

Original submission

First decision letter

MS ID#: DEVELOP/2022/201273

MS TITLE: The non-canonical Wnt receptor Ror2 is required for cartilage cell polarity and morphogenesis of the craniofacial skeleton in zebrafish

AUTHORS: Daniel B Dranow, Pierre Le Pabic, and Thomas F Schilling

I have now received two referees' reports on the above manuscript, and have reached a decision. The referees' comments are appended below, or you can access them online: please go to BenchPress and click on the 'Manuscripts with Decisions' queue in the Author Area.

As you will see, the referees express interest in your work, but feel that more work is needed to increase the novelty and impact of the work before it can be considered for publication. Both reviewers make some suggestions as to how the current experiments could be strengthened and the study extended. If you are able to revise the manuscript along the lines suggested, I will be happy receive a revised version of the manuscript.

Please also note that Development will normally permit only one round of major revision. If it would be helpful, you are welcome to contact us to discuss your revision in greater detail. Please send us a point-by-point response indicating your plans for addressing the referee’s comments, and we will look over this and provide further guidance.

Please attend to all of the reviewers' comments and ensure that you clearly highlight all changes made in the revised manuscript. Please avoid using 'Tracked changes' in Word files as these are lost in PDF conversion. I should be grateful if you would also provide a point-by-point response detailing how you have dealt with the points raised by the reviewers in the 'Response to Reviewers' box. If you do not agree with any of their criticisms or suggestions please explain clearly why this is so.

Reviewer 1

Advance summary and potential significance to field

© 2023. Published by The Company of Biologists under the terms of the Creative Commons Attribution License (https://creativecommons.org/licenses/by/4.0/).

In this study, Dranow et al. investigate the function of the non-canonical Wnt receptors Ror1 and Ror2 in cartilage morphogenesis using zebrafish models. These receptors are known to function in the planar cell polarity (PCP) pathway, which has recognized roles in skeletogenesis, particularly in chondrocyte polarity and intercalation during development and in growth plates. The goals of the project were to first determine how important Ror1/2 are for craniofacial cartilage morphogenesis, then identify potential ligands, essential functional domains, and key downstream effectors that may mediate their effects on the cytoskeleton. The authors convincingly demonstrate the redundant yet essential roles for Ror1/2 in elongation of the facial skeleton (a significant advance), and they make progress towards the other goals. However, there are weaknesses in data organization, staining quality, and the depth of their phenotypic characterization of the mutants that reduce enthusiasm for this study. Some conclusions are not strongly supported by the presented data, for example, whether the mild reduction in pFAK puncta can be attributed to a failure of ROR2 signaling through Filamin A and JNK.

Comments for the author

Fig. 1E-L: The HCR pictures are not very high quality. It seems the most important point being made here is that while ror2 is expressed in early cartilage (shown much more clearly in panel 1B), ror1 is complementarily enriched outside the condensations, especially in the jaw joint. I would remove the DAPI channel entirely and keep only panels E, H, I, L to make this point. Same comment for Fig. S1.

Fig. 2 & Lines 142-147: The images shown are maternal-zygotic mutants, but in the text they are referred to simply as mutants. The authors later explain that once they realized the mutants are viable, they maintained the line as homozygous mutants and used them for subsequent experiments, but it is confusing on the first pass. They should consider just labeling the images in the figure as “mutant” and explaining that they are MZ mutants in the legend. They also need to note somewhere whether the phenotypes of the MZ mutants are identical to those of the zygotic mutant or more severe.

Fig. 2B-C: These images are intended to show that the ror2 mutants have the “hammerhead” phenotype. This is difficult to appreciate from the dorsal images – either include markings to point out the deficit or select different images.

Fig. 2A/text: Please note whether the two ror2 mutant lines generated show the same suite of phenotypes.

Fig. 2E-F/text: As the authors note, the adult ror2 MZ mutants have a dysmorphic craniofacial skeleton – a skeletal prep of an adult specimen would be a helpful addition here – how widespread are the anomalies?

Lines 148-153: Regarding the possible pectoral fin phenotypes – these subtle differences need to be quantified and normalized to warrant inclusion in this paper. The standard lengths of the fish are given in the figure legend for the two fish shown, but juvenile fish can vary tremendously in size at 30 dpf depending on the density at which they were raised or their food intake, even without a mutation, and it is unclear how much variability is present in wild-types.

Fig. 3: The Ror2 antibody staining looks very nice and is convincingly absent in the mutant, but this information could be moved to a supplemental figure or combined with Fig. 2.

Fig. 4-5: The quantification here is not intuitive. Panels 4K-L and 5H each measure the Pq major axis/minor axis; why are they are broken out into four separate charts? Isn’t 5H the same data as in 4K and 4L? It would make more sense to have one cartilage morphology figure that combines the schematic in 5A, the images in 4A-J, one Pq major/minor graph combining 4K-M+5H, one graph for 5I (anterior neurocranium length/width) and one for 5K (Ch length/width).
Adding a quantification of the size/shape of the Sy in the different mutants would also help presage the subsequent figures focused on higher-resolution chondrocyte stacking phenotypes.

The images in 5B-G could be combined with 5J and Fig. 6 into a separate figure focused on changes in cell morphology and polarity.

© 2023. Published by The Company of Biologists under the terms of the Creative Commons Attribution License (https://creativecommons.org/licenses/by/4.0/).

Though the ror1/2 mutants clearly have a much more severe phenotype than the ror2 MZ mutants, the authors did most of their quantifications on only MZ-ror2 mutants because the double ror1/ror2 line couldn't easily be maintained as a homozygous mutant adults. They certainly seem to live to the larval stages when the analyses were done, and presumably could be generated from doubly heterozygous or homozygous/heterozygous parents. This would require more genotyping be done, but the measurements of the ror2 MZ mutants (AN L/W, Ch L/W, Tr Maj/Min) would be more valuable if they could be compared with both wild-type and double mutant fish.

The focal adhesion data is rather weak, though the experiment is better justified in the discussion than in the results section. The known links between FAs and cartilage polarity/morphogenesis and Wnt-PCP and FAs should be brought up sooner. It is premature to suggest that this small decrease in FAs in the mutant might be a direct consequence of loss of Ror2 signaling, rather than an indirect effect of impaired chondrocyte sorting. Is the effect more severe in the double ror1/ror2 mutant? In Fig. 7, it would help to outline the cluster of cells that are being quantified, otherwise the reader is distracted by the other pFAK staining elsewhere in the image.

In the rescue experiments, does the sfGFP remain attached to Ror2 after translation? Can its presence at the membrane be taken as confirmation that the different Ror2 variants are successfully making it to the membrane? Confirmation with the Ror2 antibody used in Fig. 3 that this is the case would strengthen the conclusion that the degree of rescue is indeed associated with which domains are present, not whether the variant proteins have successfully made it to the membrane.

Lines 314-315: The authors' conclusion that "ROR2's PRD mediates FAK phosphorylation through FLNA" should be toned down – the data are too circumstantial to support this statement.
Lines 317-334: The authors found that the "kinase-dead" version of zebrafish Ror2 was able to rescue chondrocyte stacking in the symplectic cartilage, implying that kinase activity is not essential for its function in this context. They also note that Ror1 is a pseudokinase lacking kinase activity and Ror2 may be as well.
However, they designed their "kinase-dead" rescue construct based on a mutation in the human ROR2 protein that "prevents ATP binding and is predicted to abolish the receptor's kinase activity in vitro". This wording implies that human ROR2 is a functional kinase, not a pseudokinase. There is nuance here about Ror2's kinase function (or lack thereof) that is not being captured.
Lines 355-359: The description of Wnt9a's interaction with Ror1/2 feels off topic. It would fit better had the authors had shown a combinatorial wnt5b;ror1;ror2 triple mutant with an enhanced phenotype (vs. resembling the wnt5b single or ror1/2 double mutants). Also, I believe "wnt5a" on line 357 is referring to the mouse gene and should be capitalized.

Line 53: Change "effect" to "affect"
Lines 83-85: Add citations
Line 137: Specify whether you are referring to the sox9a or sox9b mutant here.
Lines 390-392: Allele numbers for the new mutant lines have not been provided (i.e., just irXXXX)

<u>Reviewer 2</u>

Advance summary and potential significance to field

The manuscript "The non-canonical Wnt receptor Ror2 is required for cartilage cell polarity and morphogenesis of the craniofacial skeleton in zebrafish" by Daniel Dranow and colleagues describes a cranial cartilage phenotype in Ror2 and Ror1/Ror2 double mutant zebrafish. The mutants generated by CRISPR/Cas show aberrantly shaped cranial cartilage as well as defects in polarity and stacking of cranial chondrocytes. Additional data presented in the manuscript indicates that the Wnt-binding domain and the c-terminal proline-rich domain is required for Ror2 function in cranial cartilage, which might hint at defective WNT/PCP signaling as an underlying mechanism.
These findings are consistent with craniofacial deformations in humans affected by Recessive Robinow Syndrome and confirm observations in other organisms such as Xenopus, pigeons and mouse, thus indicating that Ror2 function in cranial cartilage morphogenesis and cartilage stacking is conserved among vertebrates.
Further, and interestingly, the authors show that chondrocytes possess fewer focal adhesions. Although this putative regulation of cell-matrix interaction and focal adhesions would uncover a

© 2023. Published by The Company of Biologists under the terms of the Creative Commons Attribution License (https://creativecommons.org/licenses/by/4.0/).

novel target of PCP downstream signaling and might shed light on the mechanistic differences between PCP signaling in epithelia and mesenchymal cells, at this stage the data is too preliminary to merit publication.

Comments for the author

Points that should be addressed prior to publication:

1) It would be essential to confirm that not only cell polarity per se as indicated by MTOC position, but indeed Wnt/PCP signaling is affected in the single and double mutants. This could be shown for example by analyzing Vangl2 localization in the chondrocytes and by testing for genetic interaction with core PCP genes such as vangl2, fzd or pk.

2) Ror2 mutants show a decrease in the number of FAK-positive focal adhesions. This might indicate towards a putative role of Ror2 and or PCP signaling in FA formation or FA signaling in chondrocytes that should be investigated further. One would need to confirm whether already focal contact formation is affected, focal adhesion maturation, dynamics or signaling and what would be the molecular signals that mediate this regulation.

3) The authors claim that Wnt5b was the most likely ligand for Ror2 and Ror1 in zebrafish. However, the manuscript does not provide any experimental confirmation. This appears even more relevant since the authors further show that the Wnt-binding CRD domain of Ror2 is required for its function in the cranial cartilage. Genetic interaction has for example been shown for wnt5b/ppt and wnt11/slb. It would be elucidating if the authors carried out similar experiments for wnt5b and ror2 and ror1 to test for genetic interaction and confirm their conclusion.

4) Based on the more severe phenotype of double null mutants, the authors conclude a partial redundancy of Ror2 and Ror1 in the cranial cartilage. This could be confirmed by expressing Ror1 in the ror2 mutant background and vice versa.

Minor points:

- The authors have analyzed cell polarity by measuring length-to-width ratio and by analyzing the position of the MTOC. The role of Ror2 and Ror1 in chondrocyte polarity could be analyzed in more detail. MTOC position should also be determined in ror1/ror2 double mutants and in a rescue situation.

- Gamma-tubulin immunofluorescence images should be shown with higher magnification and for both, WT and MZ-ror2, in Figure 6

- Higher magnification of symplectic cartilage immunofluorescence should be added to Figure 7

- Legend to Figure 4 lacks information to panels K,L and M

- “non-canonical” could be clarified as beta-catenin independent Wnt signaling

---

First revision

<u>Author response to reviewers' comments</u>

Point-by-point responses to reviewer comments

Reviewer 1 Comments for the Author:
1) Fig. 1E-L: The HCR pictures are not very high quality. It seems the most important point being made here is that while ror2 is expressed in early cartilage (shown much more clearly in panel 1B), ror1 is complementarily enriched outside the condensations, especially in the jaw joint. I would remove the DAPI channel entirely and keep only panels E, H, I, L to make

© 2023. Published by The Company of Biologists under the terms of the Creative Commons Attribution License (https://creativecommons.org/licenses/by/4.0/).

this point. Same comment for Fig. S1.

Both *ror1* and *ror2* are expressed at very low levels, which are extremely difficult to distinguish from background in traditional non-fluorescent in situs. We have repeated the HCR in situs, without much improvement in quality.
However, these patterns are extremely reproducible for *ror1* and *ror2*. In an effort to streamline the figure, as suggested by the reviewer, we have removed the all merged (DAPI, tdTomato, *ror1*, *ror2*) panels and DAPI-only panels from Figure 1 and Fig. S1. We prefer to keep the other panels.

2) Fig. 2 & Lines 142-147: The images shown are maternal-zygotic mutants, but in the text they are referred to simply as mutants. The authors later explain that once they realized the mutants are viable, they maintained the line as homozygous mutants and used them for subsequent experiments, but it is confusing on the first pass. They should consider just labeling the images in the figure as "mutant" and explaining that they are MZ mutants in the legend. They also need to note somewhere whether the phenotypes of the MZ mutants are identical to those of the zygotic mutant or more severe.

We appreciate the reviewer's constructive comments and have simplified the manuscript and figures by referring to maternal-zygotic ror2-/- mutants simply as "*ror2*$^{-/-}$" throughout and providing further explanation in the manuscript text (lines 134-139).

3) Fig. 2B-C: These images are intended to show that the ror2 mutants have the "hammerhead" phenotype. This is difficult to appreciate from the dorsal images – either include markings to point out the deficit or select different images.

A "hammerhead" like-phenotype in zebrafish simply refers to craniofacial mutants with a general reduction of tissue anterior to the eyes that causes the eyes to appear to protrude (this mutant class was initially described in the forward genetic mutant screens in Tubingen). We have included markings in Figure 2, panels B-C' to make this more apparent.

4) Fig. 2A/text: Please note whether the two ror2 mutant lines generated show the same suite of phenotypes.

We now note in the revised manuscript (lines136-137) that the second *ror2* mutant line (-4 bp deletion)^{ir1094} displays a phenotype identical to the one used throughout the study (-1 bp deletion)^{ir1093}. We also describe that we have not noticed a difference between zygotic or maternal-zygotic mutants (lines 136-139).

5) Fig. 2E-F/text: As the authors note, the adult ror2 MZ mutants have a dysmorphic craniofacial skeleton – a skeletal prep of an adult specimen would be a helpful addition here – how widespread are the anomalies?

We now include several examples of alizarin red stained adult *ror2* mutants to illustrate the overall dysmorphic craniofacial skeleton, as well as indicating several specific bones with readily apparent differing morphologies in new Figure S3.

6) Lines 148-153: Regarding the possible pectoral fin phenotypes – these subtle differences need to be quantified and normalized to warrant inclusion in this paper. The standard lengths of the fish are given in the figure legend for the two fish shown, but juvenile fish can vary tremendously in size at 30 dpf depending on the density at which they were raised or their food intake, even without a mutation, and it is unclear how much variability is present in wild-types.

We now include in Figure S4 quantification of length-to-width ratios of endoskeletal discs (eds) from 16 WT animals (32 eds) and 14 *ror2*$^{-/-}$ mutants (28 eds), which confirms a significant difference ($p<0.001$). We use eye diameter rather than standard length for staging samples as *ror2* mutant embryos/larvae are shorter than WT (as quantified for 5 dpf larva in Fig.2 D). We now specifically mention why we use eye diameter to normalize in the legend of Fig. S4.

© 2023. Published by The Company of Biologists under the terms of the Creative Commons Attribution License (https://creativecommons.org/licenses/by/4.0/).

7) Fig. 3: The Ror2 antibody staining looks very nice and is convincingly absent in the mutant, but this information could be moved to a supplemental figure or combined with Fig. 2.

We have moved this to Supplemental Figures as suggested, now called Fig. S5

8) Fig. 4-5: The quantification here is not intuitive. Panels 4K-L and 5H each measure the Pq major axis/minor axis; why are they are broken out into four separate charts? Isn't 5H the same data as in 4K and 4L? It would make more sense to have one cartilage morphology figure that combines the schematic in 5A, the images in 4A-J, one Pq major/minor graph combining 4K-M+5H, one graph for 5I (anterior neurocranium length/width) and one for 5K (Ch length/width).
Adding a quantification of the size/shape of the Sy in the different mutants would also help presage the subsequent figures focused on higher-resolution chondrocyte stacking phenotypes.

We thank the reviewer for this suggestion. We now include similar quantifications of symplectic cartilages comparing several genotypes in Figure 3 and described in lines 169-172 of the results section and lines 528-532 of the methods.

9) The images in 5B-G could be combined with 5J and Fig. 6 into a separate figure focused on changes in cell morphology and polarity.

We have combined the higher resolution cell shape images and trabecular cell shape quantification with the gamma- tubulin cell polarity data in Figure 4.

10) Though the ror1/2 mutants clearly have a much more severe phenotype than the ror2 MZ mutants, the authors did most of their quantifications on only MZ-ror2 mutants because the double ror1/ror2 line couldn't easily be maintained as a homozygous mutant adults. They certainly seem to live to the larval stages when the analyses were done, and presumably could be generated from doubly heterozygous or homozygous/heterozygous parents. This would require more genotyping be done, but the measurements of the ror2 MZ mutants (AN L/W, Ch L/W, Tr Maj/Min) would be more valuable if they could be compared with both wild-type and double mutant fish.

We have now included length-to-width ratio measurements for sy, pq, ch cartilages and anterior neurocrania for 4 additional genotypes: *ror1*$^{-/-}$, *ror1*$^{-/-}$; ror2$^{-/-}$, *wnt5b*$^{-/-}$, and *ror2*$^{-/-}$; *wnt5b*$^{-/-}$ in Figure 3.

11) The focal adhesion data is rather weak, though the experiment is better justified in the discussion than in the results section. The known links between FAs and cartilage polarity/morphogenesis and Wnt-PCP and FAs should be brought up sooner. It is premature to suggest that this small decrease in FAs in the mutant might be a direct consequence of loss of Ror2 signaling, rather than an indirect effect of impaired chondrocyte sorting. Is the effect more severe in the double ror1/ror2 mutant? In Fig. 7, it would help to outline the cluster of cells that are being quantified, otherwise the reader is distracted by the other pFAK staining elsewhere in the image.

We now specifically mention "cell polarity" in our rationale for examining FAs in the results section (line 237).
We agree with the reviewer that it is difficult to uncouple a decrease in cell adhesion/FAs and cell polarity without more direct mechanistic evidence. Unfortunately, we do not have the tools to distinguish between a model in which aberrant Ror2 signaling cause alterations in cell polarity and as a consequence changes/reductions in cell adhesion or one in which defective signaling cause a reduction in cell adhesion/FAs which contribute to a cell polarity phenotype.
We now outline the symplectic cartilages in which pFAK foci were quantified in Figure 5.

12) In the rescue experiments, does the sfGFP remain attached to Ror2 after translation? Can its presence at the membrane be taken as confirmation that the different Ror2 variants are successfully making it to the membrane? Confirmation with the Ror2 antibody used in

© 2023. Published by The Company of Biologists under the terms of the Creative Commons Attribution License (https://creativecommons.org/licenses/by/4.0/).

Fig. 3 that this is the case would strengthen the conclusion that the degree of rescue is indeed associated with which domains are present, not whether the variant proteins have successfully made it to the membrane.

sfGFP remains attached to Ror2 following translation. All Ror2 rescue constructs are fusion proteins engineered to result in the production of a single RNA and protein (e.g., for full-length Ror2, ror2 without its stop codon was amplified by PCR and gibson assembly performed to yield a sequence in-frame with a small flexible linker peptide and sfGFP. A single RNA encoding a single protein is produced from the construct. sfGFP on its own would be targeted to the cytoplasm while Ror2 would be targeted to the membrane. Therefore, as the reviewer suggests, GFP at the membrane is indicative of Ror2 rescue constructs properly localizing to the membrane. Only the *ror2* portions of the rescue construct contain the native sequences necessary for membrane targeting.

13) Lines 314-315: The authors' conclusion that "ROR2's PRD mediates FAK phosphorylation through FLNA" should be toned down – the data are too circumstantial to support this statement.

We have toned this down by moving the section on the FA data later in the Discussion and adjusting the text as follows: "What is the mechanistic relationship between Ror2 and FA formation? WNT5A-bound ROR2 activates c-Jun N-terminal kinase (JNK), a direct activator of FAK, in wound-healing assays in a FLNA-dependent manner (Nomachi et al., 2008), **however additional studies are required to directly test whether ROR2's PRD mediates FAK phosphorylation through FLNA."** (Lines 372-374).

14) Lines 317-334: The authors found that the "kinase-dead" version of zebrafish Ror2 was able to rescue chondrocyte stacking in the symplectic cartilage, implying that kinase activity is not essential for its function in this context. They also note that Ror1 is a pseudokinase lacking kinase activity and Ror2 may be as well. However, they designed their "kinase-dead" rescue construct based on a mutation in the human ROR2 protein that "prevents ATP binding and is predicted to abolish the receptor's kinase activity in vitro". This wording implies that human ROR2 is a functional kinase, not a pseudokinase. There is nuance here about Ror2's kinase function (or lack thereof) that is not being captured.

We have tried to tighten-up the language to explain this more clearly. Ror1 is referred to as a pseudokinase as there is no experimental evidence demonstrating that it has any in vitro kinase activity. In addition, it shares certain features of its kinase domain with other RTKs that lack kinase activity or that have been experimentally tested to abolish kinase activity in other RTK family members. The kinase domains of RTKs are very highly conserved and decades of research in several other RTKs has elucidated which residues in this highly conserved domain are necessary for in vitro kinase activity. We now say:

RESULTS: "Transgenic constructs included full-length Ror2 (Ror2FL), Ror2 with a mutation **predicted to render any RTK kinase domain inactive (kinase-dead mutation; Ror2KD)**…"(Lines 262-263)

DISCUSSION: "…we attempted to rescue cartilage stacking defects in zebrafish through the mosaic expression of a **predicted** kinase-dead Ror2 variant. The K507R mutation in human **ROR2 targets a highly conserved residue in the kinase domain of RTKs that prevents ATP binding and abolishes an RTK receptor's kinase activity in vitro (**Matsuda et al., 2003)." (Lines 302-304)

An additional point of confusion is that based on previous evidence from their research group, Matsuda and colleagues (2003), believed that Ror2 had an active kinase domain.

15) Lines 355-359: The description of Wnt9a's interaction with Ror1/2 feels off topic. It would fit better had the authors had shown a combinatorial wnt5b;ror1;ror2 triple mutant with an enhanced phenotype (vs. resembling the wnt5b single or ror1/2 double mutants). Also, I believe "wnt5a" on line 357 is referring to the mouse gene and should be capitalized.

© 2023. Published by The Company of Biologists under the terms of the Creative Commons Attribution License (https://creativecommons.org/licenses/by/4.0/).

We chose to keep the discussion of *Wnt9a* genetic interactions with *Ror1* and *Ror2* and studies of the zebrafish *wnt9a* mutants as it emphasizes the conserved and distinct features of the skeletal phenotypes detected in each species.

We have produced *ror2;wnt5b* double mutants and have included analysis of length-to-width ratios of several cartilages to Figure 3. Please see our response to Reviewer 2, point (3) for additional explanation. We agree that it would be interesting to analyze the phenotypes of additional double and possibly triple mutant combinations.
Unfortunately, due to the difficulty in obtaining sufficient numbers of animals it would not have been feasible for us to produce *wnt5b;ror1;ror2* triple mutants or other mutant combinations in the limited time we had for revision. However, given the phenotype of *ror2;wnt5b* double mutants, we suspect that *wnt5b;ror1;ror2* triple mutants would not have a significantly more severe phenotype.

We thank the reviewer for their attention to detail; indeed, we were referring to the mouse gene and have capitalized to Wnt9a.

16) Line 53: Change “effect” to “affect”

Done.

17) Lines 83-85: Add citations

Added citation for review by (Endo et al., 2015) (Lines 86-87)

18) Line 137: Specify whether you are referring to the sox9a or sox9b mutant here.

We now specify *sox9a*$^{-/-}$.

19) Lines 390-392: Allele numbers for the new mutant lines have not been provided (i.e., just irXXXX)

We have now included allele numbers for *ror1* and *ror2* alleles in the methods section and Figure S2.

Reviewer 2 Comments for the Author:
Points that should be addressed prior to publication:

1) It would be essential to confirm that not only cell polarity per se as indicated by MTOC position, but indeed Wnt/PCP signaling is affected in the single and double mutants. This could be shown for example by analyzing Vangl2 localization in the chondrocytes and by testing for genetic interaction with core PCP genes such as vangl2, fzd or pk.

We have attempted to address this with numerous approaches. However, this is challenging in zebrafish due to a lack of tools to examine localization of these proteins including the absence of zebrafish-specific antibodies and transgenic tools. To date, most tools used to examine PCP component localization in zebrafish involve the injection of RNA encoding GFP-tagged versions of these proteins, which do not persist to the developmental stages that we examine here. Though Vangl2 localization is polarized to the proximal side of chondrocytes in the developing mouse limb, Ror2 is not (Gao et al., 2011), and no group has shown that any core PCP proteins are asymmetrically localized in zebrafish cartilages. Additionally, while the MTOC position/polarity within developing long bones of chick and mouse (Li et al. 2009) resembles our findings in zebrafish craniofacial cartilages (Le Pabic et al., 2014), asymmetric localization of Vangl2 has not been reported in chick chondrocytes (Li et al. 2009).

We tried zebrafish-specific polyclonal antibodies raised against Vangl2 (Genetex GTX54230) and Prickle1a (Genetex GTX54227), but while these stained epithelial membranes during gastrulation, they only appeared to stain muscles at 55 and 72 hpf. We also examined Vangl2 localization in cartilage using transgenic *Tg(vangl2:EGFP-vangl2)*^{zou002} animals (Sittaramane et al. 2013). Using

© 2023. Published by The Company of Biologists under the terms of the Creative Commons Attribution License (https://creativecommons.org/licenses/by/4.0/).

an anti-GFP antibody, we found that EGFP-Vangl2 localized to the membranes of chondrocytes in the craniofacial skeleton and strongly in the oral epithelium, but we did not observe any asymmetric localization of EGFP-Vangl2, similar to chick. Because zebrafish chondrocytes are so closely adherent and not separated by significant ECM at 3 days postfertilization, we thought that we may have not been able to detect asymmetric EGFP-Vangl2. To address this possibility, we performed neural crest cell transplants from *Tg(vangl2:EGFP-vangl2)^{zou002}* donors into *Tg(sox10:lyn-tdtomato)* hosts to label subsets of cartilage cells, but still did not observe obvious asymmetries. Additional transplants and a more extensive analysis would be required to draw any conclusions. Below, we have included examples of *vangl2:EGFP* expression in 3 dpf *Tg(vangl2:EGFP- vangl2)^{zou002}* animals (A) as well as an example of *Tg(vangl2:EGFP-vangl2)^{zou002}* donor into *(sox10:lyn-tdTomato)* host neural crest cell transplants in the ceratohyal (B, B').

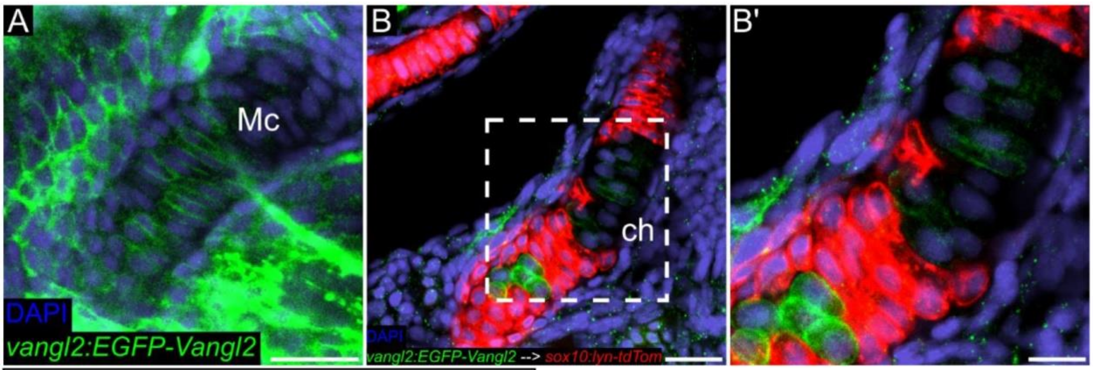

Scale bars = 20 μm for A and B, 10 μm for B'.

To examine genetic interactions with other core PCP genes, we produced *ror2$^{-/-}$; vangl2$^{-/-}$* double mutants and while we think these animals may have more severe cartilage phenotypes than *ror2$^{-/-}$* mutants (at least in the pq cartilage), problems with the alcian staining quality of these samples prevented us from documenting and quantifying these data. Unfortunately, we did not have enough time to repeat the crosses, genotyping, staining protocol, and perform the analysis before the revision deadline. …

References cited in this response:

Gao B, Song H, Bishop K, Elliot G, Garrett L, English MA, Andre P, Robinson J, Sood R, Minami Y, Economides AN, Yang Y. Wnt signaling gradients establish planar cell polarity by inducing Vangl2 phosphorylation through Ror2. Dev Cell. 2011 Feb 15;20(2):163-76. doi: 10.1016/j.devcel.2011.01.001. PMID: 21316585; PMCID: PMC3062198.

Li Y, Junge JA, Arnesano C, Gross GG, Miner JH, Moats R, Roberts RW, Arnold DB, Fraser SE. Discs large 1 controls daughter-cell polarity after cytokinesis in vertebrate morphogenesis. Proc Natl Acad Sci U S A. 2018 Nov 13;115(46):E10859-E10868. doi: 10.1073/pnas.1713959115. Epub 2018 Oct 30. PMID: 30377270; PMCID: PMC6243286.

Li Y, Dudley AT. Noncanonical frizzled signaling regulates cell polarity of growth plate chondrocytes. Development. 2009 Apr;136(7):1083-92. doi: 10.1242/dev.023820. Epub 2009 Feb 18. PMID: 19224985; PMCID: PMC2685929.

Sittaramane V, Pan X, Glasco DM, Huang P, Gurung S, Bock A, Li S, Wang H, Kawakami K, Matise MP, Chandrasekhar A. The PCP protein Vangl2 regulates migration of hindbrain motor neurons by acting in floor plate cells, and independently of cilia function. Dev Biol. 2013 Oct 15;382(2):400-12. doi: 10.1016/j.ydbio.2013.08.017. Epub 2013 Aug 26. PMID: 23988578; PMCID: PMC3864009.

2) Ror2 mutants show a decrease in the number of FAK-positive focal adhesions. This might indicate towards a putative role of Ror2 and or PCP signaling in FA formation or FA signaling in

© 2023. Published by The Company of Biologists under the terms of the Creative Commons Attribution License (https://creativecommons.org/licenses/by/4.0/).

chondrocytes that should be investigated further. One would need to confirm whether already focal contact formation is affected, focal adhesion maturation, dynamics or signaling and what would be the molecular signals that mediate this regulation.

To address this, we performed pFAK antibody staining at a stage during which chondrocytes are actively stacking, however we, encountered several issues which prevented us from being able to perform an extensive analysis of the data. First, prechondrocytes in the symplectic cartilage condensation are very tightly packed together in 3- dimensional space, making it very difficult to distinguish one cell from another and to localize pFAK foci to them. We attempted to count all pFAK foci within the entire condensation and found a difference between *ror2*$^{-/-}$ and WT but not one that was statistically significant.

As an alternative approach to investigate the role of focal adhesions further in the context of cartilage stacking, we attempted to disrupt FA regulation during craniofacial morphogenesis using a FAK Inhibitor 14 (FAK14; Cayman Chemical 14485), an inhibitor of FAK1 autophosphorylation. We treated embryos during between ~50-72 hpf, when craniofacial cartilages are stacking, at concentrations of 25, 50, 75, 100, 150, and 200 µM inhibitor (from 50 mM stock dissolved in DMSO) in embryo medium. We found that larvae appeared phenotypically normal at concentrations of 25-75 µM FAK14 and had hypoplastic but otherwise well-stacked craniofacial cartilages at higher concentrations (100-200 µM). All larva survived in treatments of 25-100 uM FAK14 but we observed ~10% and >50% lethality at 15 and 200 µM FAK14, respectively. Therefore, we were unfortunately unable to use this inhibitor to uncouple formation of pFAK foci from Wnt-Ror cell polarity.

3) The authors claim that Wnt5b was the most likely ligand for Ror2 and Ror1 in zebrafish. However, the manuscript does not provide any experimental confirmation. This appears even more relevant since the authors further show that the Wnt-binding CRD domain of Ror2 is required for its function in the cranial cartilage. Genetic interaction has for example been shown for wnt5b/ppt and wnt11/slb. It would be elucidating if the authors carried out similar experiments for wnt5b and ror2 and ror1 to test for genetic interaction and confirm their conclusion.

To examine genetic interactions, we produced *ror2*$^{-/-}$; *wnt5b*$^{-/-}$ double mutants and now include our analysis of these animals in the revised manuscript (Figure 3K, L, M-P). Briefly, we found that *ror2*$^{-/-}$; *wnt5b*$^{-/-}$ were not significantly different from *wnt5b*$^{-/-}$ alone (Fig. 3 M-P). This result is consistent with Wnt5b being the primary ligand for Ror1 and Ror2 receptors in zebrafish craniofacial cartilages.

4) Based on the more severe phenotype of double null mutants, the authors conclude a partial redundancy of Ror2 and Ror1 in the cranial cartilage. This could be confirmed by expressing Ror1 in the ror2 mutant background and vice versa.

We thank the reviewer for this suggestion. To test this, we produced a full-length zebrafish Ror1-sfGFP fusion protein construct similar to our Ror2 full-length rescue construct, *sox10:ror1-sfGFP*. We injected this construct into *ror2*$^{-/-}$ mutants and found that Ror1-sfGFP it further rescued symplectic cartilage stacking/length to an extent (Figure 6N-O). As the *ror1* mutant has no apparent phenotype on its own, it seems unlikely that injection of a Ror2 full-length construct would yield any result of interest so this was not attempted.

Minor points:

- The authors have analyzed cell polarity by measuring length-to-width ratio and by analyzing the position of the MTOC. The role of Ror2 and Ror1 in chondrocyte polarity could be analyzed in more detail. MTOC position should also be determined in ror1/ror2 double mutants and in a rescue situation.

Thank you for the suggestion. Since there were no significant differences in symplectic cartilage length-to-width ratios (Figure 3M) and MTOC positions appeared to be equally/randomly distributed in the sy (Fig. 4N), we do not think analysis of *ror1; ror2* double mutants or attempts at rescuing them would yield any new insights. However, a comparison of MTOC position between

© 2023. Published by The Company of Biologists under the terms of the Creative Commons Attribution License (https://creativecommons.org/licenses/by/4.0/).

cartilages with differing severity between *ror2* and *ror1; ror2* mutants (such as the palatoquadrate) could yield more interesting results. However, despite our attempts it was extremely difficult to produce enough *ror1;ror2* mutants for experiments and the numbers required for analysis of MTOC polarity in cartilages. We agree that it would be interesting to examine MTOC polarity in all craniofacial cartilages and across multiple genotypes, but could not obtain sufficient double mutant animals for such analyses.

- Gamma-tubulin immunofluorescence images should be shown with higher magnification and for both, WT and MZ- ror2, in Figure 6

We have added higher magnification views of Gamma-tubulin antibody-stained WT and *ror2*$^{-/-}$ mutant symplectic cartilages (Figure 4. K', L').

- Higher magnification of symplectic cartilage immunofluorescence should be added to Figure 7

We chose to stick with the original magnification because individual cells are visible and it is helpful to be able to see it in the context of the whole organ shape.

- Legend to Figure 4 lacks information to panels K,L and M

We thank the reviewer for noticing this oversight. We have included this missing information in the revised manuscript (Figure 3 legend).

- "non-canonical" could be clarified as beta-catenin independent Wnt signaling

We have specified that "non-canonical" refers to beta-catenin independent Wnt signaling in the abstract and introduction (Line 18, Line 45): "Non-canonical / β-catenin-independent Wnt signaling plays critical roles…" Also mentioned in line 86 of the discussion.

Second decision letter

MS ID#: DEVELOP/2022/201273

MS TITLE: The non-canonical Wnt receptor Ror2 is required for cartilage cell polarity and morphogenesis of the craniofacial skeleton in zebrafish

AUTHORS: Daniel B Dranow, Pierre Le Pabic, and Thomas F Schilling

I have now received all the referees reports on the above manuscript, and have reached a decision. The referees' comments are appended below, or you can access them online: please go to BenchPressand click on the 'Manuscripts with Decisions' queue in the Author Area.

There are some minor issues raised in the reviews that you should consider/address before we proceed to publication. Reviewer 1 has some corrections and reviewer 2 would like you to ensure that you don't overstate the conclusions on mechanism that can be drawn from your data.

Reviewer 1

Advance summary and potential significance to field

In this study, Dranow et al. investigate the function of the non-canonical Wnt receptors Ror1 and Ror2 in cartilage morphogenesis using zebrafish models. These receptors are known to function in the non-canonical Wnt planar cell polarity (PCP) pathway, which has recognized roles in skeletogenesis and growth plates. The goals of the project were to determine how important Ror1/2 are for craniofacial cartilage morphogenesis, then identify potential ligands, essential functional domains, and key downstream effectors that may mediate their effects on the

© 2023. Published by The Company of Biologists under the terms of the Creative Commons Attribution License (https://creativecommons.org/licenses/by/4.0/).

cytoskeleton. The authors convincingly demonstrate the redundant roles for Ror2 and to a lesser degree Ror1 in elongation of facial cartilages and identify Wnt5b as a likely ligand. They further show that the Ror2 proline-rich domain and the extracellular Wnt-binding domain are necessary for rescue of the mutant phenotype while the kinase function is not, adding to accumulating evidence that Ror2 is a pseudokinase. Finally, they show that chondrocytes of mutant cartilages are less polarized and have fewer focal adhesion puncta compared to controls, suggesting a possible mechanism by which Wnt-PCP regulates cartilage morphogenesis.

Comments for the author

I am very satisfied with the changes the authors have made to the manuscript and figures.

(very) minor comments:

Line 157: Change “confirming that ror2 is a likely null mutant” to “confirming that our mutant alleles are likely
null”, in order to distinguish between the gene name and the mutant line.

Line 231: Delete “cell” after “cytoskeleton”.

Line 362: The abbreviation GP has not been defined.

Lines 371-2: Delete the extra “mediates” after PRD, and add a period to the end of the sentence.

Line 524: Change “Sy cartilage” to “Sy cartilages”

Line 571: refers to panels A-L but Figure only goes up to J.

Line 591: delete the extra period

<u>Reviewer 2</u>

Advance summary and potential significance to field

The manuscript “The non-canonical Wnt receptor Ror2 is required for cartilage cell polarity and morphogenesis of the craniofacial skeleton in zebrafish” by Daniel Dranow and colleagues describes a cranial cartilage phenotype in Ror2 and Ror1/Ror2 double mutant zebrafish. The mutants generated by CRISPR/Cas show aberrantly shaped cranial cartilage as well as defects in polarity and stacking of cranial chondrocytes. Additional data presented in the manuscript indicates that the Wnt-binding domain and the c-terminal proline-rich domain is required for Ror2 function in cranial cartilage, which might hint at defective WNT/PCP signaling as an underlying mechanism.
These findings are consistent with craniofacial deformations in humans affected by Recessive Robinow Syndrome and confirm observations in other organisms such as Xenopus, pigeons and mouse, thus indicating that Ror2 function in cranial cartilage morphogenesis and cartilage stacking is conserved among vertebrates.

In the revised version, the authors further show that Wnt5b-deficient larvae show a stronger phenotype than Ror2 single mutant and that Wnt5b/Ror2 double mutants are similar to Wnt5a single mutants. These observations confirm Wnt5b as the predominant ligand of Ror1/Ror2.

Comments for the author

The manuscript considerably benefited from the additional experiments and data added, in particular quantification of phenotypes and the genetic interaction with Wnt5b and the partial rescue by Ror1. These confirm a Wnt5b/ Ror1/ Ror2 axis in cartilage morphogenesis and show that there is some but not full redundancy between Ror1 and Ror2.

I strongly appreciate the efforts the authors went through to address the questions raised in my review of the first version of the manuscript. Unfortunately, though, not all experiments the authors attempted were successful in the sense that they could contribute to elucidating the

© 2023. Published by The Company of Biologists under the terms of the Creative Commons Attribution License (https://creativecommons.org/licenses/by/4.0/).

molecular mechanisms underlying Ror2 function in zebrafish cranial cartilage. Therefore, the conclusion that the observed polarity defects and reduced number of focal adhesions are mechanistically linked to Ror2 function in Wnt/PCP signaling is still not sufficiently supported by experimental evidence. I understand that addressing the molecular mechanisms is experimentally very challenging and time consuming. However, it should be stated clearly that the study in these points is merely descriptive and the interpretation and potential mechanism discussed with references to the relevant original publications that support it.

Second revision

Author response to reviewers' comments

Responses to reviewer comments

Reviewer 1 Comments for the Author:

I am very satisfied with the changes the authors have made to the manuscript and figures.

(very) minor comments:

Line 157: Change “confirming that ror2 is a likely null mutant” to “confirming that our mutant alleles are likely null”, in order to distinguish between the gene name and the mutant line.
Done.

Line 231: Delete “cell” after “cytoskeleton”.
Done.

Line 362: The abbreviation GP has not been defined.
We have added the abbreviation to the introduction, line 71. “Similarly, Wnt-PCP signaling regulates cartilage polarity in the developing growth plates (GPs) of mammalian long bones (Kuss et al., 2014).”

Lines 371-2: Delete the extra “mediates” after PRD, and add a period to the end of the sentence.
Done.

Line 524: Change “Sy cartilage” to “Sy cartilages”
Done.

Line 571: refers to panels A-L but Figure only goes up to J.
Fixed.

Line 591: delete the extra period
Done.

Reviewer 2 Comments for the Author:

The manuscript considerably benefited from the additional experiments and data added, in particular quantification of phenotypes and the genetic interaction with Wnt5b and the partial rescue by Ror1. These confirm a Wnt5b/ Ror1/ Ror2 axis in cartilage morphogenesis and show that there is some but not full redundancy between Ror1 and Ror2.

I strongly appreciate the efforts the authors went through to address the questions raised in my review of the first version of the manuscript. Unfortunately, though, not all experiments the

© 2023. Published by The Company of Biologists under the terms of the Creative Commons Attribution License (https://creativecommons.org/licenses/by/4.0/).

authors attempted were successful in the sense that they could contribute to elucidating the molecular mechanisms underlying Ror2 function in zebrafish cranial cartilage. Therefore, the conclusion that the observed polarity defects and reduced number of focal adhesions are mechanistically linked to Ror2 function in Wnt/PCP signaling is still not sufficiently supported by experimental evidence. I understand that addressing the molecular mechanisms is experimentally very challenging and time consuming. However, it should be stated clearly that the study in these points is merely descriptive and the interpretation and potential mechanism discussed with references to the relevant original publications that support it.

We have added to the discussion (lines 372-374): “Alternatively, the reduction in FAs in *ror2* mutants might be a secondary consequence of a more general loss of cell polarity.”

Third decision letter

MS ID#: DEVELOP/2022/201273

MS TITLE: The non-canonical Wnt receptor Ror2 is required for cartilage cell polarity and morphogenesis of the craniofacial skeleton in zebrafish

AUTHORS: Daniel B Dranow, Pierre Le Pabic, and Thomas F Schilling
ARTICLE TYPE: Research Articles

I am happy to tell you that your manuscript has been accepted for publication in Development, pending our standard ethics checks.

© 2023. Published by The Company of Biologists under the terms of the Creative Commons Attribution License (https://creativecommons.org/licenses/by/4.0/).
